# Supplementary material for: A framework for conducting GWAS using repeated measures data with an application to childhood BMI
Source: Nat Commun. 2024 Nov 20;15:10067. doi: 10.1038/s41467-024-53687-3 (PMC11579382; doi:10.1038/s41467-024-53687-3)
Supplement: Supplementary file 1 — Supplementary Information [file 41467_2024_53687_MOESM1_ESM.pdf]

Supplementary Figures and Notes for:

A framework for conducting GWAS using repeated measures data with an application to BMI across childhood

Supplementary Figure 1: Flow diagram of analysis pipeline.. 3
Supplementary Figure 2: Refinement of knot points in the cubic spline with cubic slope random effects model 4
A: ALSPAC 4
B: CHOP-EA 5
C: CHOP-AA 6
D: NFBC1966.. 7
E: NFBC1986.. 8
F: OBE 9
Supplementary Figure 3: Residual plots for the chosen model 10
1: ALSPAC.. 10
2: CHOP-EA 12
3: CHOP-AA 14
4: NFBC1966 16
5: NFBC1986 18
6: OBE 20
Supplementary Figure 4: Scatter plots of the estimated phenotypes from our preferred model compared to estimated phenotypes from a model also including a continuous autoregressive correlation structure of order 1 (CAR(1)) for males and females in the ALSPAC and NFBC1986 cohorts... 22
ALSPAC – Males: 22
ALSPAC – Females: 23
NFBC1986 - Males.. 24
NFBC1986 – Females:.. 25
Supplementary Figure 5: Scatter plots of the estimated phenotypes from our preferred model compared to estimated phenotypes from a model including a cubic spline function in the random effects and a continuous autoregressive correlation structure of order 1 (CAR(1)) for males and females in the ALSPAC and NFBC1986 cohorts 26
ALSPAC – Males: 26
ALSPAC – Females: 27
NFBC1986 – Males:.. 28
NFBC1986 – Females:.. 29
Supplementary Figure 6: Summary of the association between final BMI (between age 16-18 years) and each of the estimated phenotypes, including a random effects meta-analysis (DerSimonian-Laird estimator) 30
Supplementary Figure 7: Quantile-quantile (QQ) plots of the heterogeneity test in the European meta-analyses for the estimated phenotypes 33
Supplementary Figure 8: Quantile-quantile (QQ) plots of the heterogeneity test in the European meta-analyses for the estimated phenotypes, excluding the OBE cohort. 34

Supplementary Figure 9: Manhattan plots of the meta-analyses without OBE for the phenotypes across infancy (0-0.5 years), early childhood (1.5-3.5 years), late childhood (6.5-10 years) and adolescence (12-17 years) **35**
Supplementary Figure 10: LocusZoom regional plot of the association between adolescent slope and the FAM120AOS locus **38**
Supplementary Note 1: Membership of the Early Growth Genetics (EGG) Consortium 39
Supplementary Note 2: Cohort description, acknowledgements and funding 43
Avon Longitudinal Study of Parents and Children (ALSPAC) 43
Northern Finland Birth Cohorts born in 1966 (NFBC1966) 44
Northern Finland Birth Cohorts born in 1986 (NFBC1986) 45
CHOP 46
OBE 46
Supplementary Note 3: Example process and output from the *EGGLA* BMI modelling framework 46
Supplementary Note 4: Linear mixed modelling 48
Supplementary References 49

Supplementary Figures

Supplementary Figure 1: Flow diagram of analysis pipeline

The flow diagram illustrates the step by step procedure used to implement, test, assess and finalize non-linear BMI curve modeling ready for GWAS. Future analysts may consult this flow diagram and read further information in the Supplementary Note 4 about the outputs from the EGGLA pipeline.

*ALSPAC Avon Longitudinal Study of Parents and Children; CHOP-EA Children's Hospital of Philadelphia European subset; CHOP-AA Children's Hospital of Philadelphia African American subset; NFBC1966 Northern Finland Birth Cohort 1966; NFBC1986 Northern Finland Birth Cohort 1986; LMM linear mixed model; GWAS genome-wide association study; BMI body mass index; glm generalized linear model; PCs principal components; QC quality control. *Here model refinement is specific to the present BMI growth analysis. For other growth methods, model refinement will be specific to the chosen model and knot refinements for spline models may not be appropriate.*

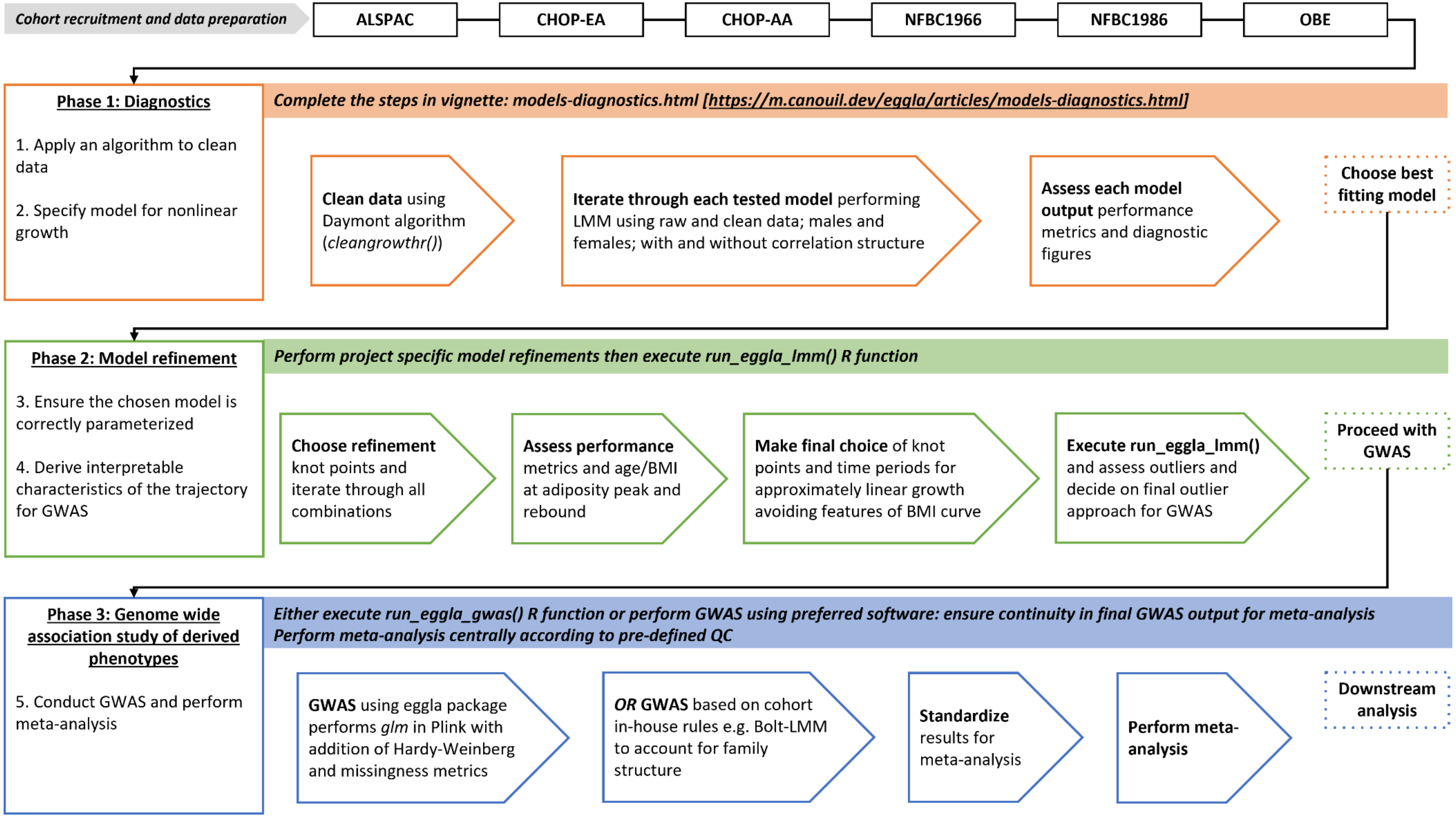

Supplementary Figure 2: Refinement of knot points in the cubic spline with cubic slope random effects model

The cubic spline with cubic slope random effects models were selected for refinement. Models iterated pairwise through increments of knot 1 (1, 1.5 and 2 years) and knot 2 (6, 7, and 8 years), with knot 3 being held constant at 12 years. The figure displays the performance metrics AIC, conditional R^2, and RMSE as a function of average age at adiposity peak and rebound. The knot point combinations are differentially coloured for knot 1 (yellow for 1 year, blue for 1.5 years, and green for 2 years) and has a differential marker symbol to denote knot 2 (circle for 6 years, square for 7 years, and triangle for 8 years). Marker colour indicates males (blue) and females (pink). Figures are given for ALSPAC [A](N=7197 males and 6818 females), CHOP-EA [B] (N= 12002 males and 10814 females), CHOP-AA [C](N= 10533 males and 10772 females), NFBC1966 [D] (N= 3800 males and 3280 females), NFBC1986 [E] (N= 2734 males and 2826 females), and OBE [F] (N= 252 males and 308 females).

ALSPAC Avon Longitudinal Study of Parents and Children; CHOP-EA Children's Hospital of Philadelphia European subset; CHOP-AA Children's Hospital of Philadelphia African American subset; NFBC1966 Northern Finland Birth Cohort 1966; NFBC1986 Northern Finland Birth Cohort 1986; AIC Akaike information criterion ; RMSE root mean square error.

A: ALSPAC

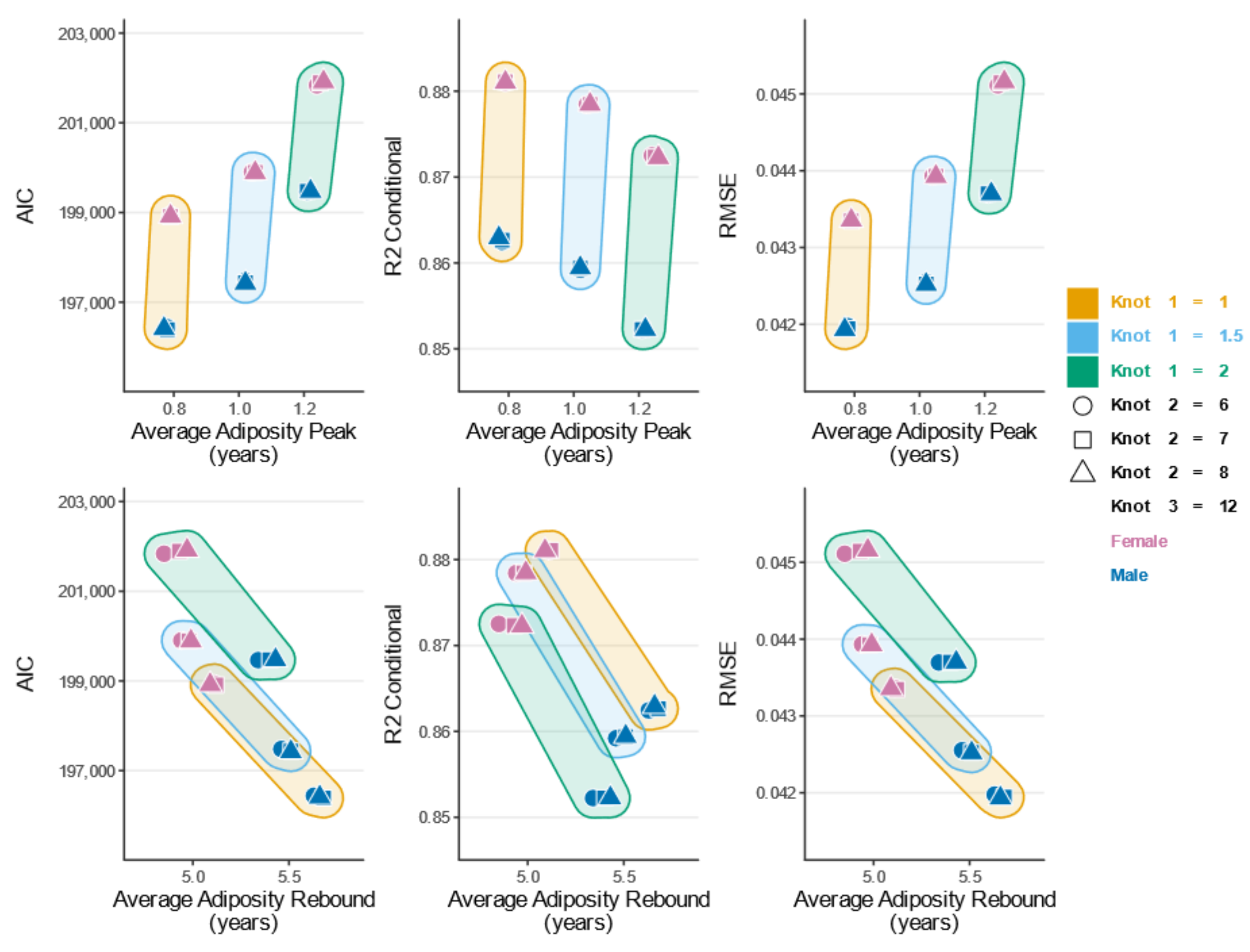

B: CHOP-EA

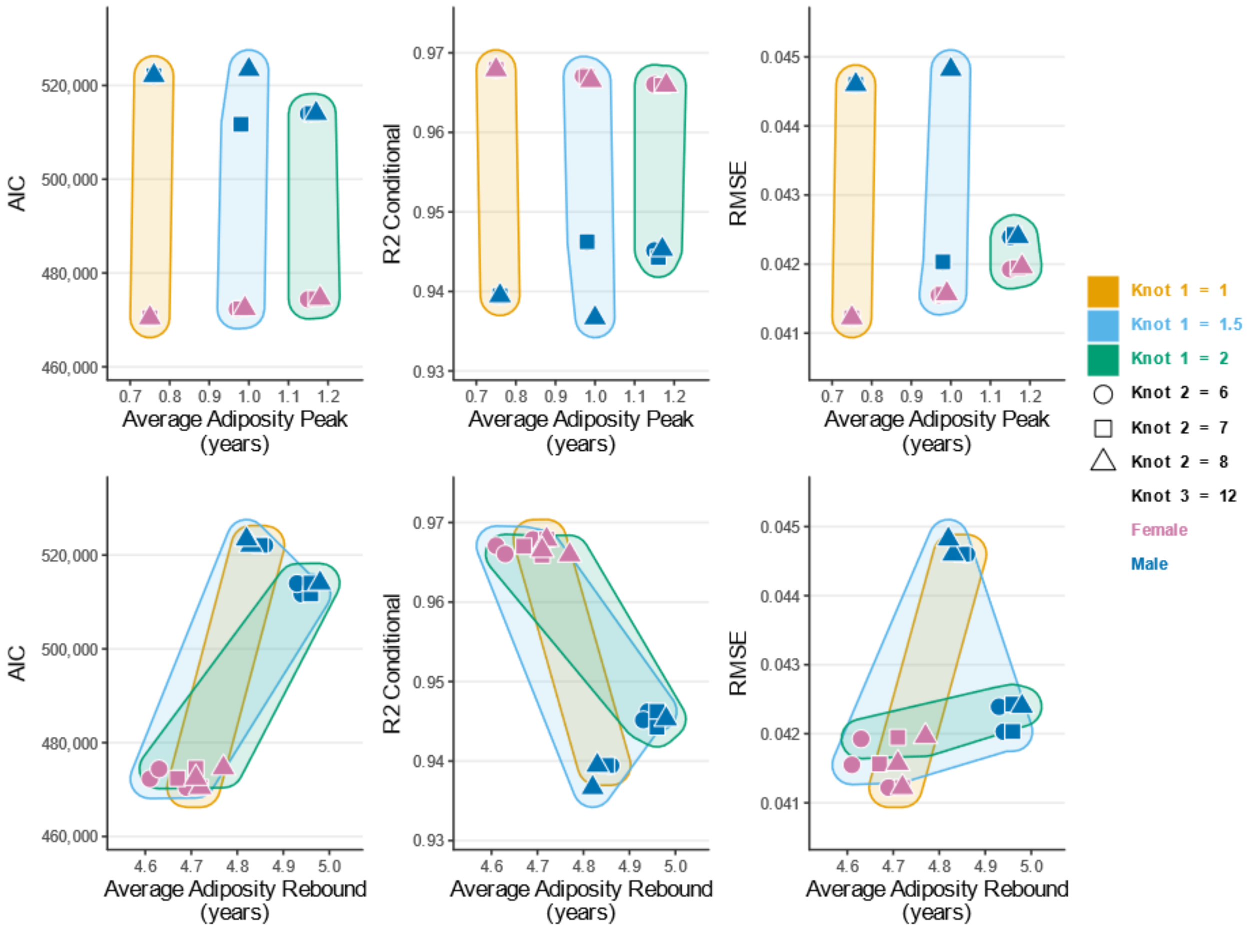

AIC
R2 Conditional
RMSE
Average Adiposity Peak (years)
Average Adiposity Rebound (years)
Knot 1 = 1
Knot 1 = 1.5
Knot 1 = 2
Knot 2 = 6
Knot 2 = 7
Knot 2 = 8
Knot 3 = 12
Female
Male

C: CHOP-AA

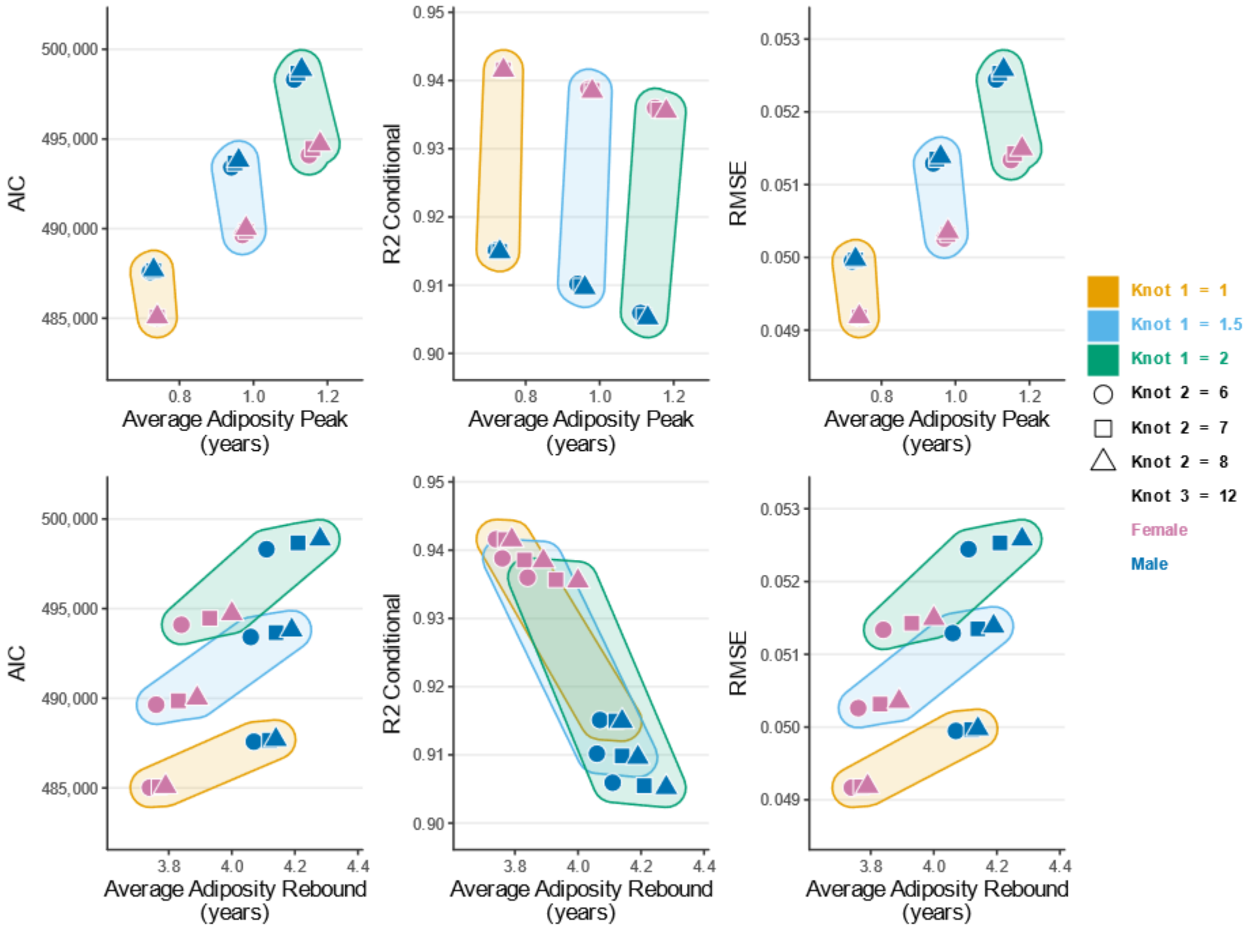
AIC
R2 Conditional
RMSE
Average Adiposity Peak (years)
Average Adiposity Rebound (years)
Knot 1 = 1
Knot 1 = 1.5
Knot 1 = 2
Knot 2 = 6
Knot 2 = 7
Knot 2 = 8
Knot 3 = 12
Female
Male

D: NFBC1966

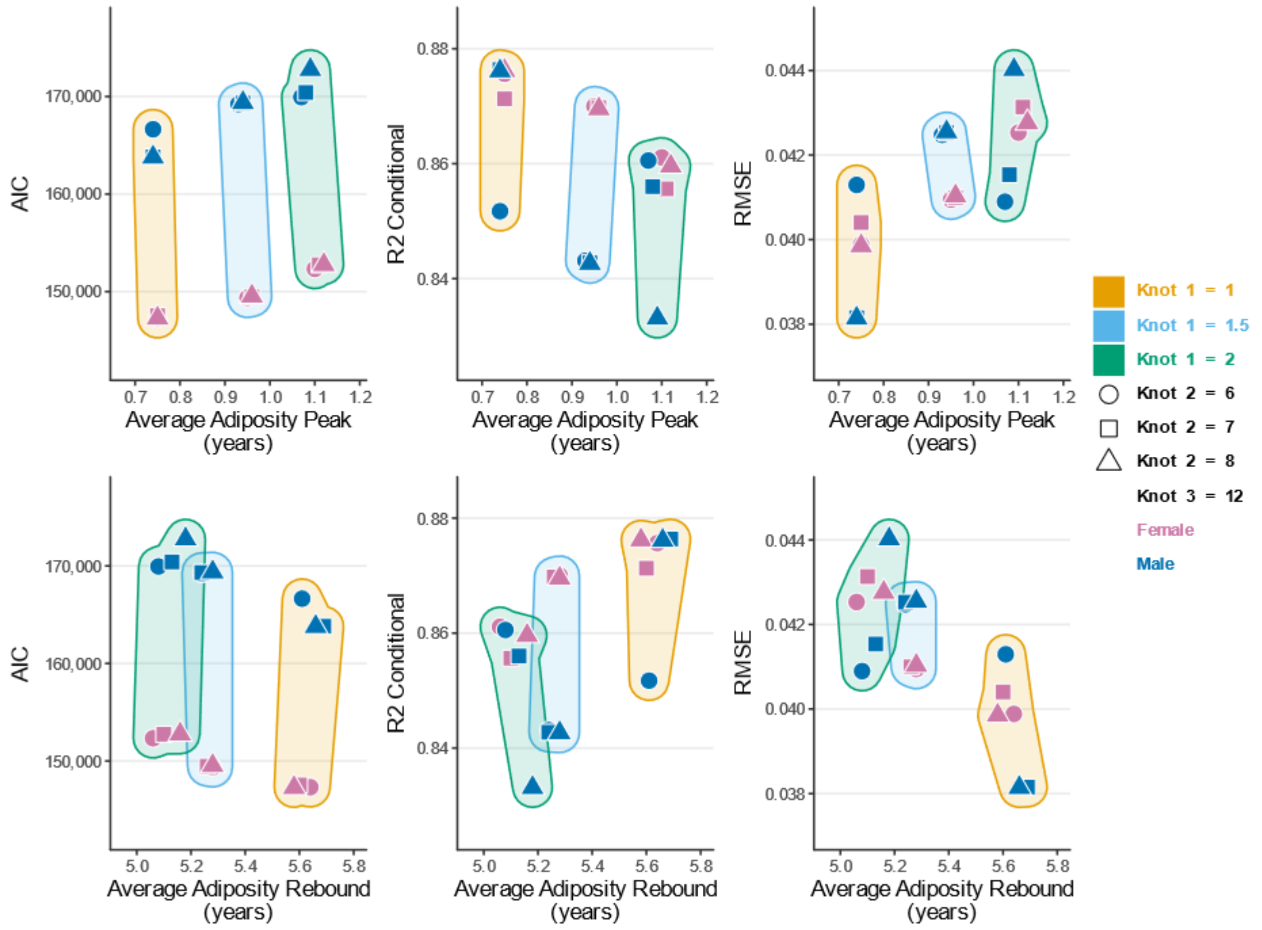
AIC
170,000
160,000
150,000
Average Adiposity Peak (years)
R2 Conditional
0.88
0.86
0.84
RMSE
0.044
0.042
0.040
0.038
0.7 0.8 0.9 1.0 1.1 1.2
Average Adiposity Rebound (years)
5.0 5.2 5.4 5.6 5.8
Knot 1 = 1
Knot 1 = 1.5
Knot 1 = 2
Knot 2 = 6
Knot 2 = 7
Knot 2 = 8
Knot 3 = 12
Female
Male

E: NFBC1986

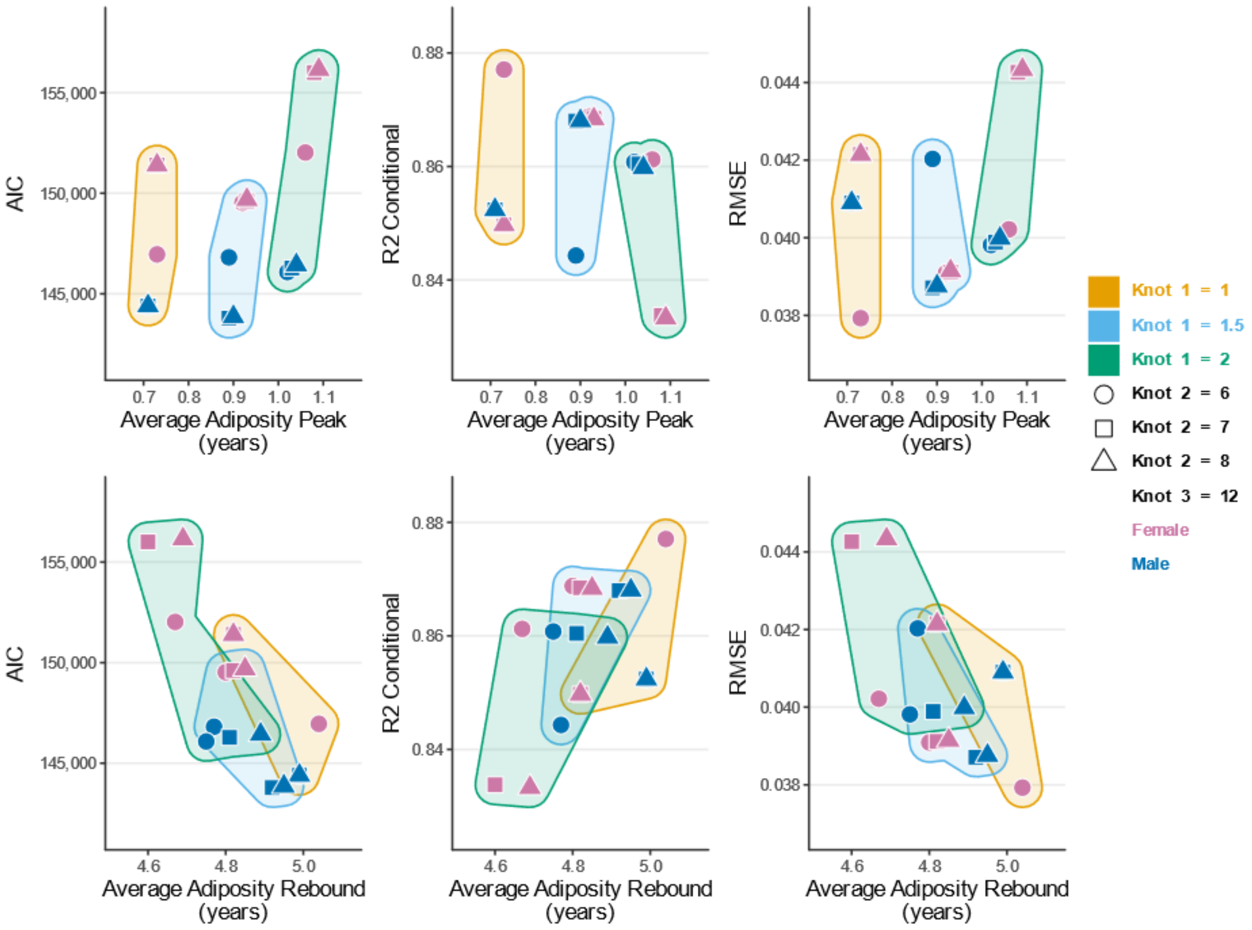

AIC
155,000
150,000
145,000
0.7
0.8
0.9
1.0
1.1
Average Adiposity Peak (years)
R2 Conditional
0.88
0.86
0.84
RMSE
0.044
0.042
0.040
0.038
4.6
4.8
5.0
Average Adiposity Rebound (years)
Knot 1 = 1
Knot 1 = 1.5
Knot 1 = 2
Knot 2 = 6
Knot 2 = 7
Knot 2 = 8
Knot 3 = 12
Female
Male

F: OBE

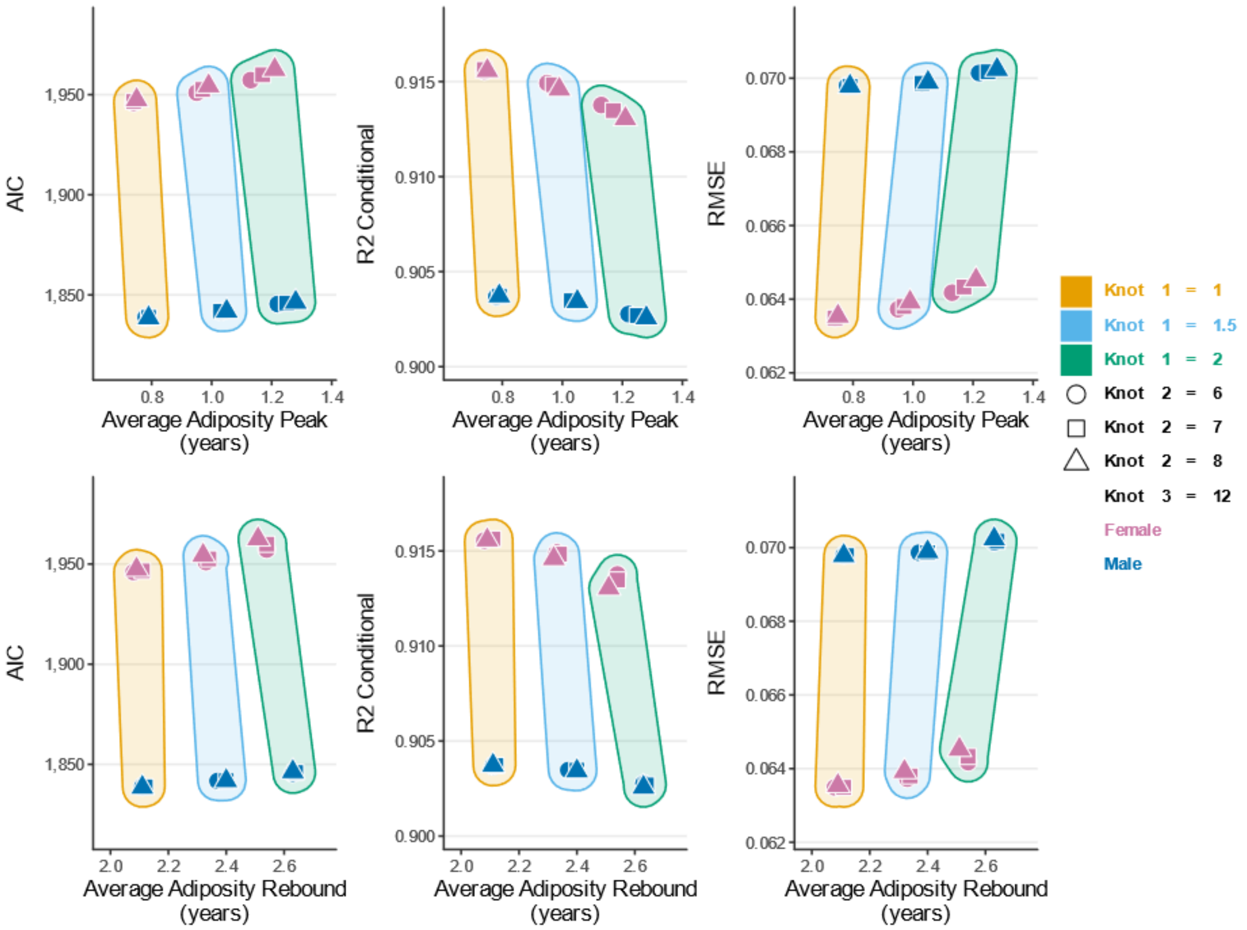

AIC
1,950
1,900
1,850
0.8
1.0
1.2
1.4
Average Adiposity Peak (years)
R2 Conditional
0.915
0.910
0.905
0.900
RMSE
0.070
0.068
0.066
0.064
0.062
Average Adiposity Rebound (years)
2.0
2.2
2.4
2.6
Knot 1 = 1
Knot 1 = 1.5
Knot 1 = 2
Knot 2 = 6
Knot 2 = 7
Knot 2 = 8
Knot 3 = 12
Female
Male

Supplementary Figure 3: Residual plots for the chosen model

Assessment of model fit for our final chosen model (cubic spline with cubic slope random effects). Each plot depicts: A) a scatter plot of the fitted BMI values vs. Observed (the orange line is the x=y line) B) a scatter plot of the fitted BMI values vs. marginal residuals (the orange line is the mean value) C) a scatter plot of the fitted age values vs. marginal residuals (the orange line is y=0) D) autocorrelation function (ACF) vs. lag of normalized residuals (the blue dashed line is y=0) E) a QQ plot of the theoretical vs. sample quantiles (the blue dashed line is the expected value) and F) a QQ plot of the theoretical vs. residual quantiles (the blue dashed line is the expected value). 1) ALSPAC (N=7197 males and 6818 females); 2) CHOP-EA (N= 12002 males and 10814 females); 3) CHOP-AA (N= 10533 males and 10772 females) 4) NFBC1966 (N= 3800 males and 3280 females); 5) NFBC1986 (N= 2734 males and 2826 females); 6) OBE (N= 252 males and 308 females).

ALSPAC Avon Longitudinal Study of Parents and Children; CHOP-EA Children's Hospital of Philadelphia European subset; CHOP-AA Children's Hospital of Philadelphia African American subset; NFBC1966 Northern Finland Birth Cohort 1966; NFBC1986 Northern Finland Birth Cohort 1986

1: ALSPAC

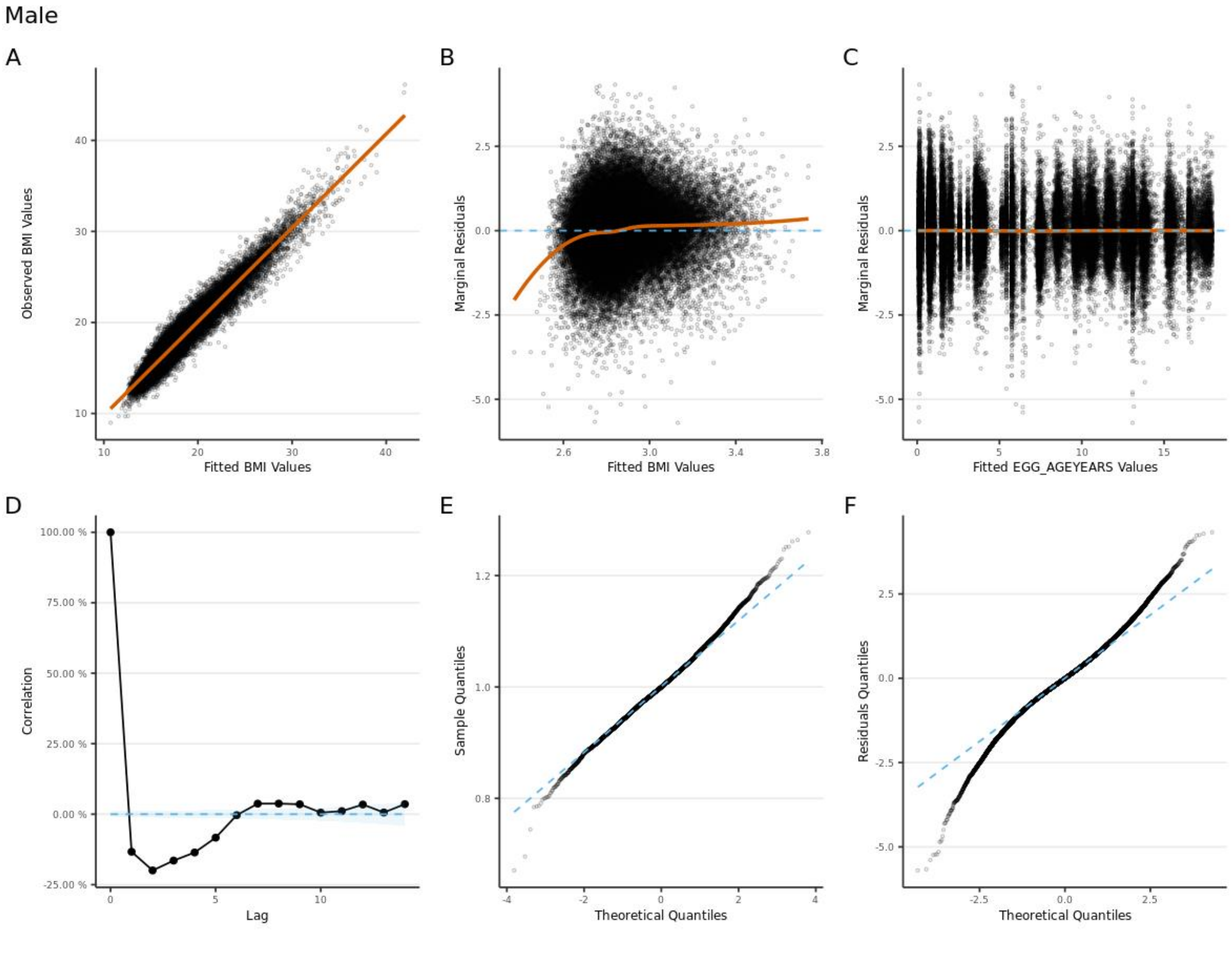

Female

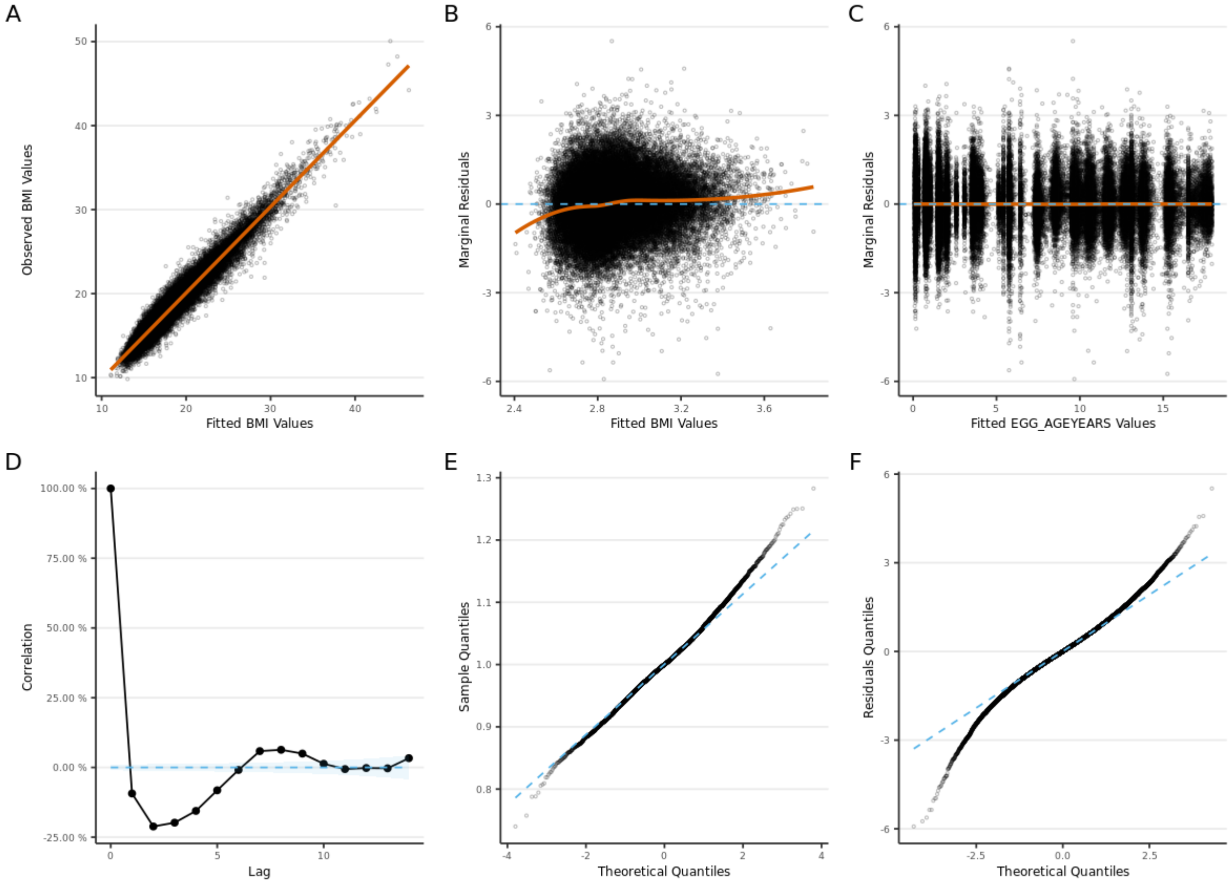
A
Observed BMI Values
Fitted BMI Values
B
Marginal Residuals
Fitted BMI Values
C
Marginal Residuals
Fitted EGG_AGEYEARS Values
D
Correlation
Lag
E
Sample Quantiles
Theoretical Quantiles
F
Residuals Quantiles
Theoretical Quantiles

2: CHOP-EA

Male

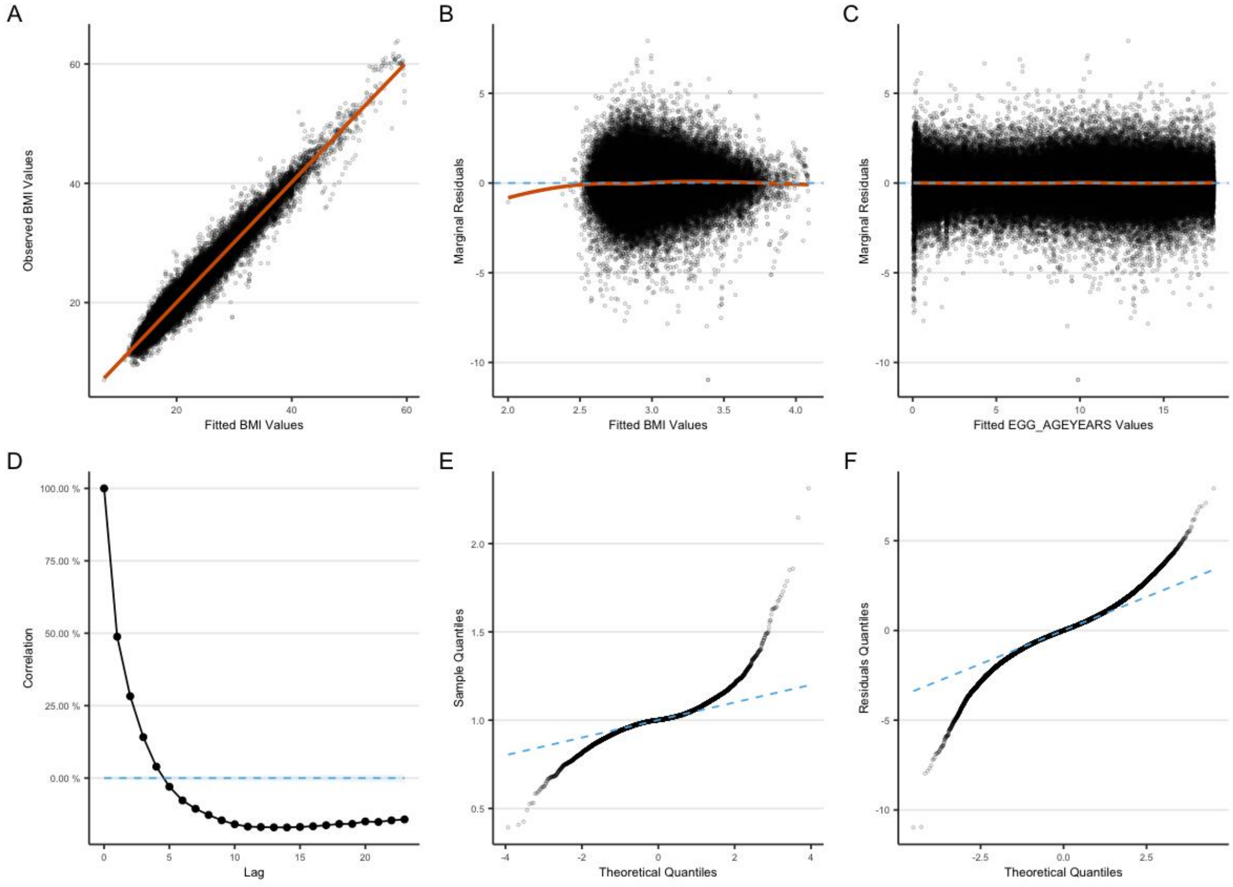

Female

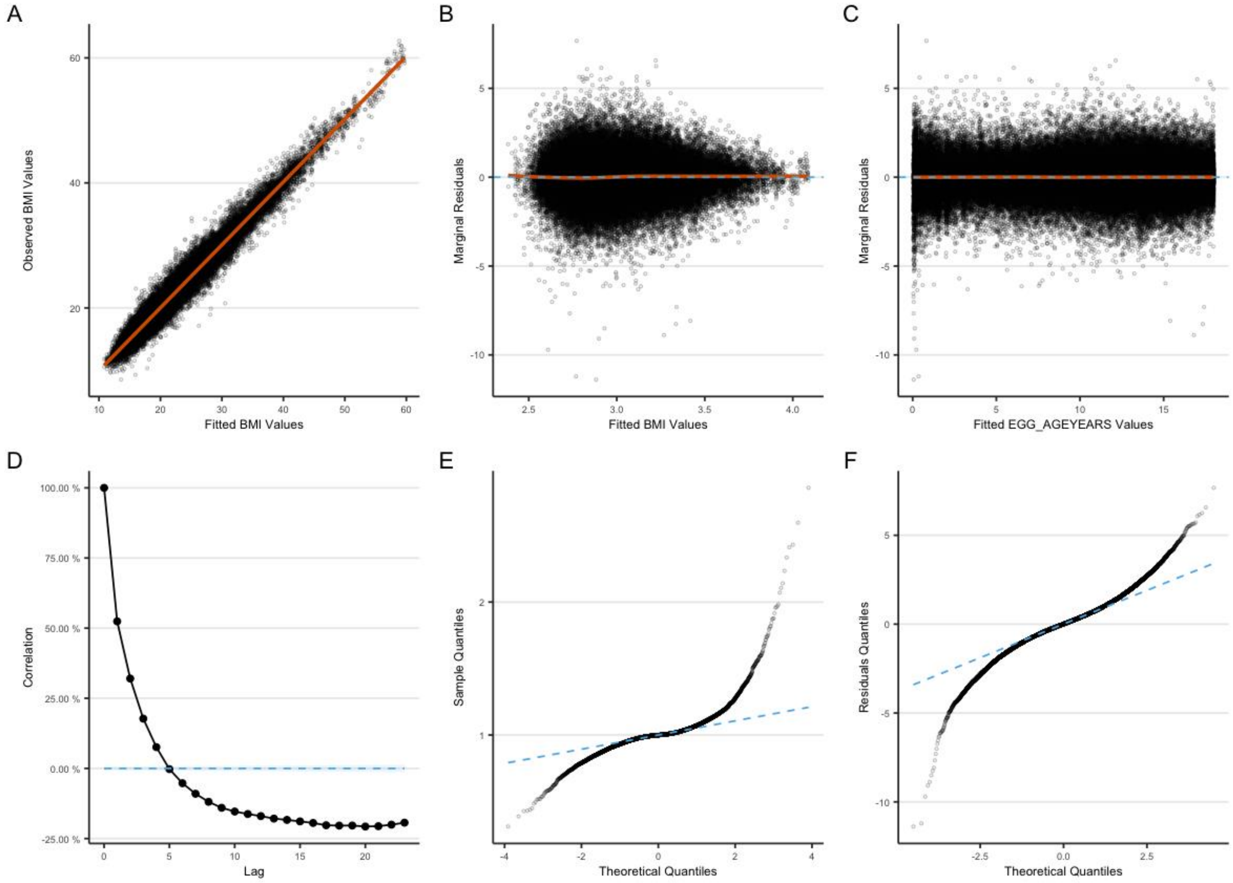

A
Observed BMI Values
Fitted BMI Values
B
Marginal Residuals
Fitted BMI Values
C
Marginal Residuals
Fitted EGG_AGEYEARS Values
D
Correlation
Lag
E
Sample Quantiles
Theoretical Quantiles
F
Residuals Quantiles
Theoretical Quantiles

3: CHOP-AA

Male

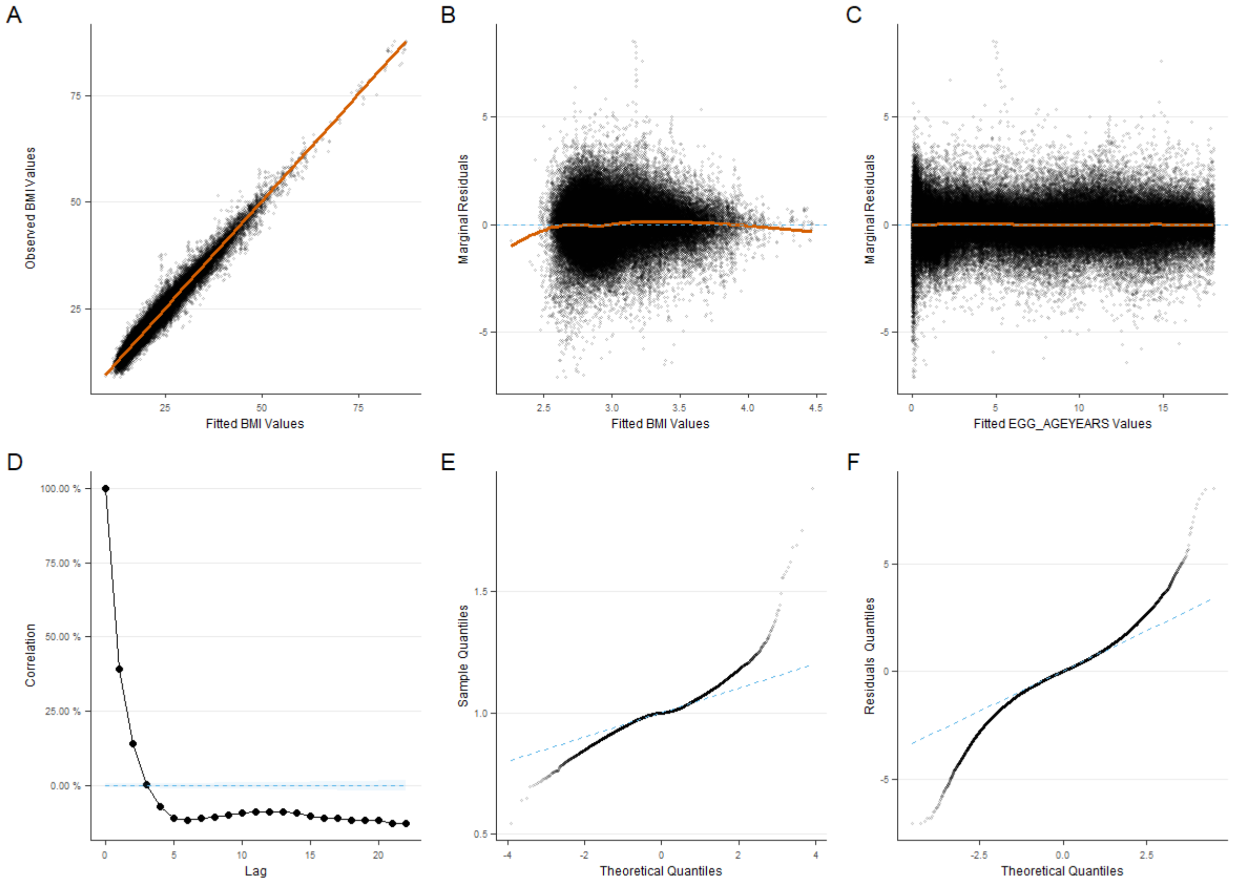

Female

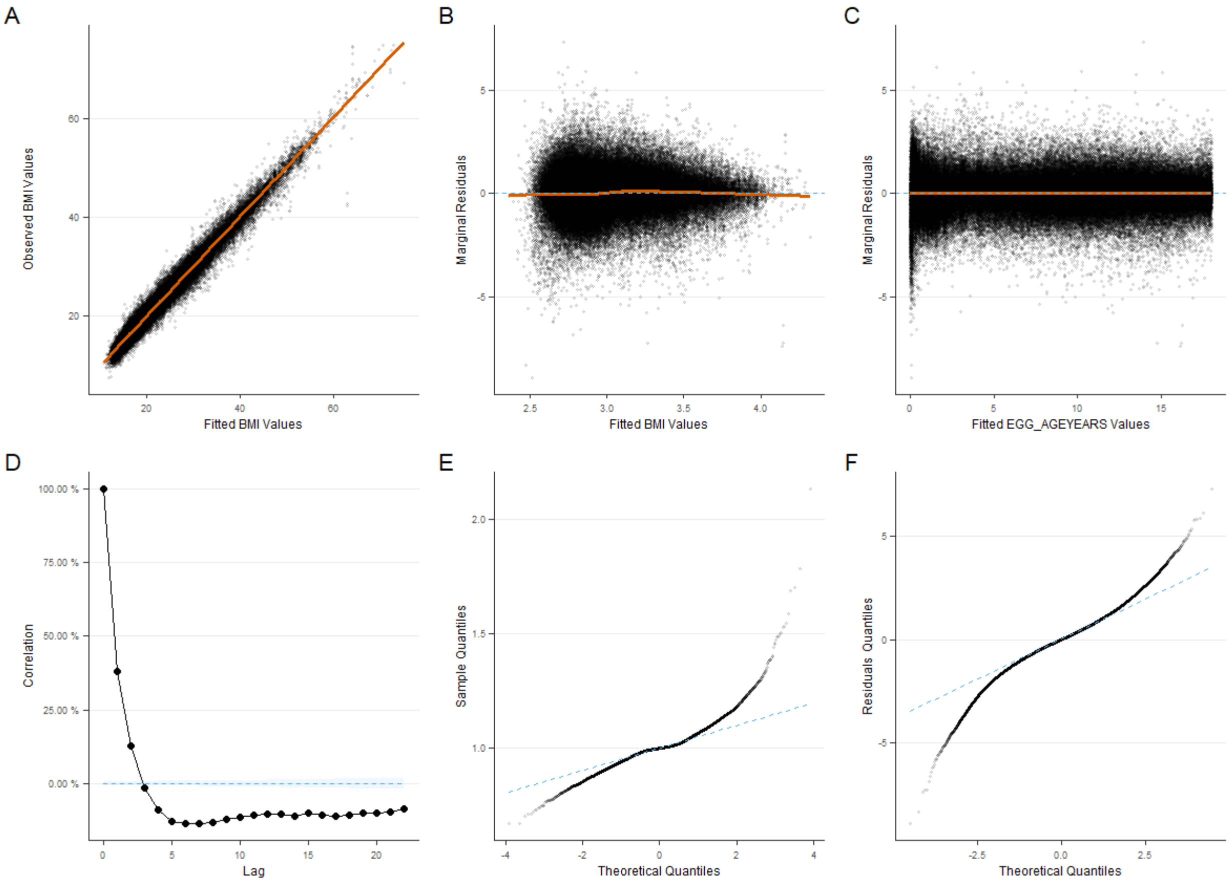
A
B
C
D
E
F
Observed BMI Values
Fitted BMI Values
Marginal Residuals
Fitted BMI Values
Marginal Residuals
Fitted EGG_AGEYEARS Values
Correlation
Lag
Sample Quantiles
Theoretical Quantiles
Residuals Quantiles
Theoretical Quantiles

4: NFBC1966

Male

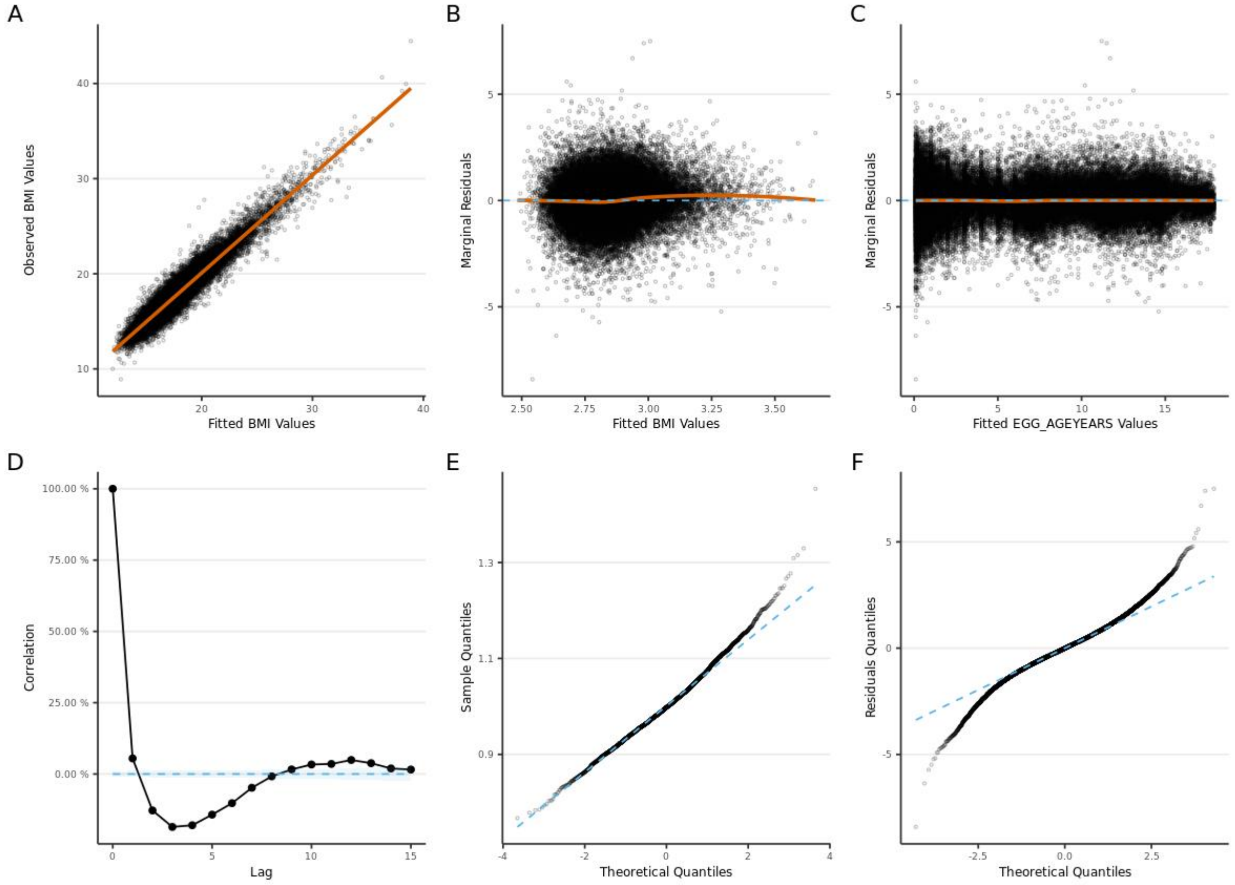

Female

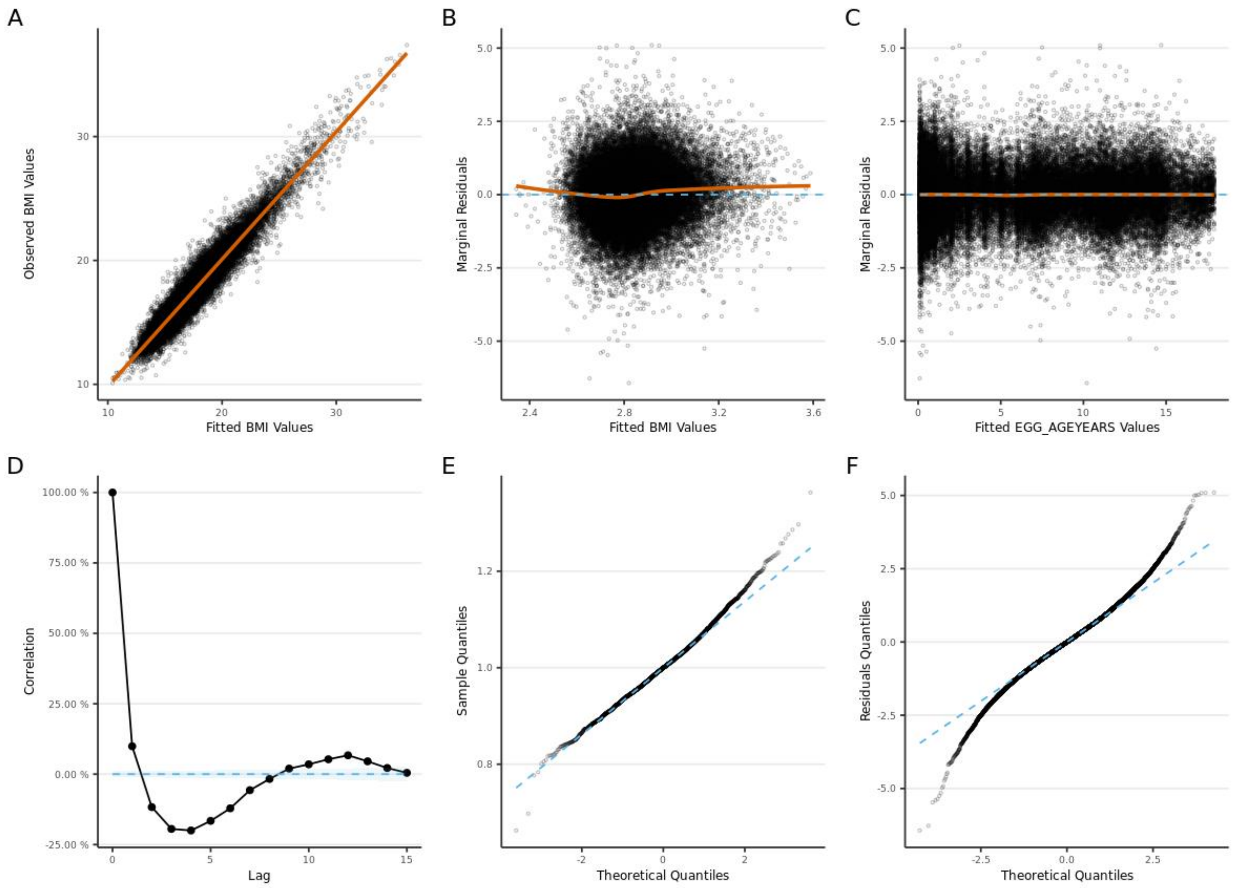
A
B
C
D
E
F
Observed BMI Values
Fitted BMI Values
Marginal Residuals
Fitted EGG_AGEYEARS Values
Correlation
Lag
Sample Quantiles
Theoretical Quantiles
Residuals Quantiles

5: NFBC1986

Male

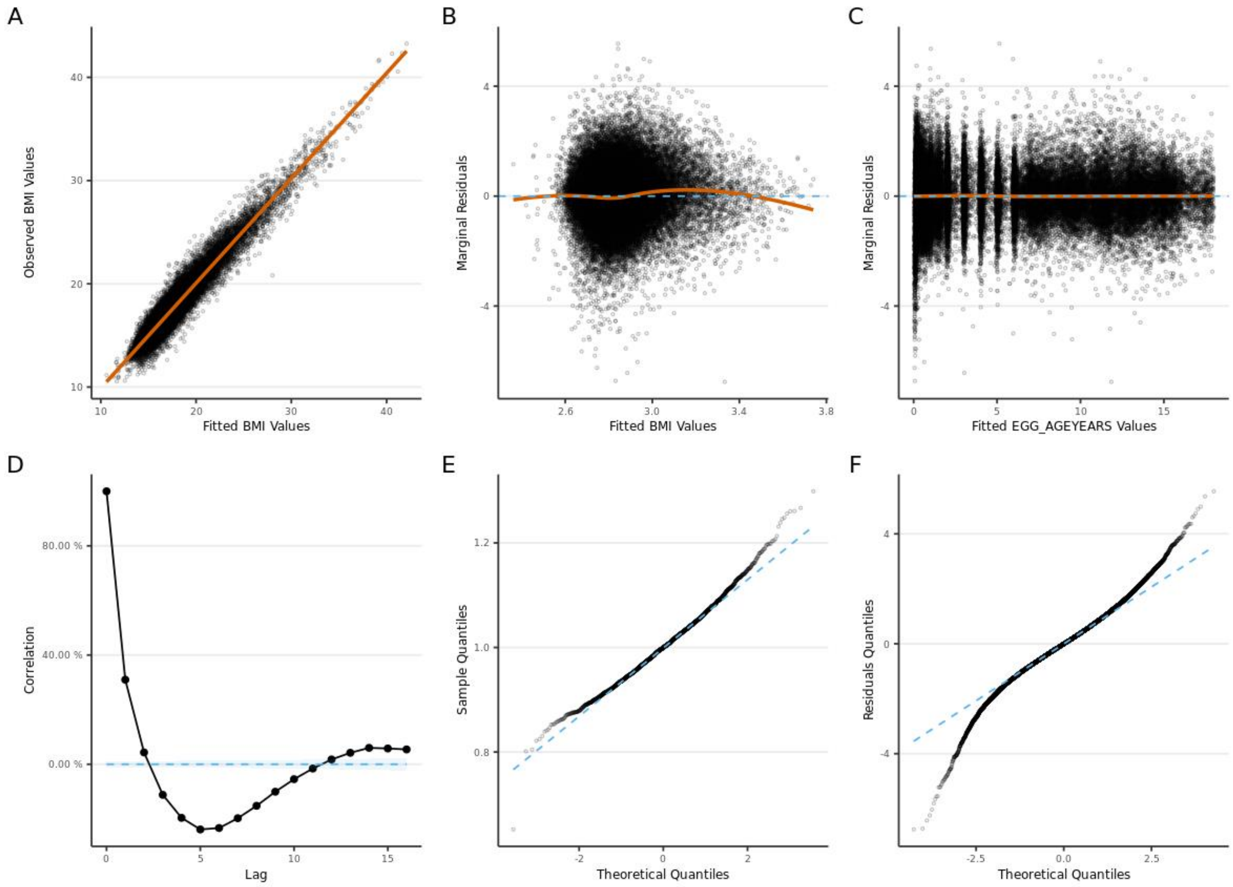

Female

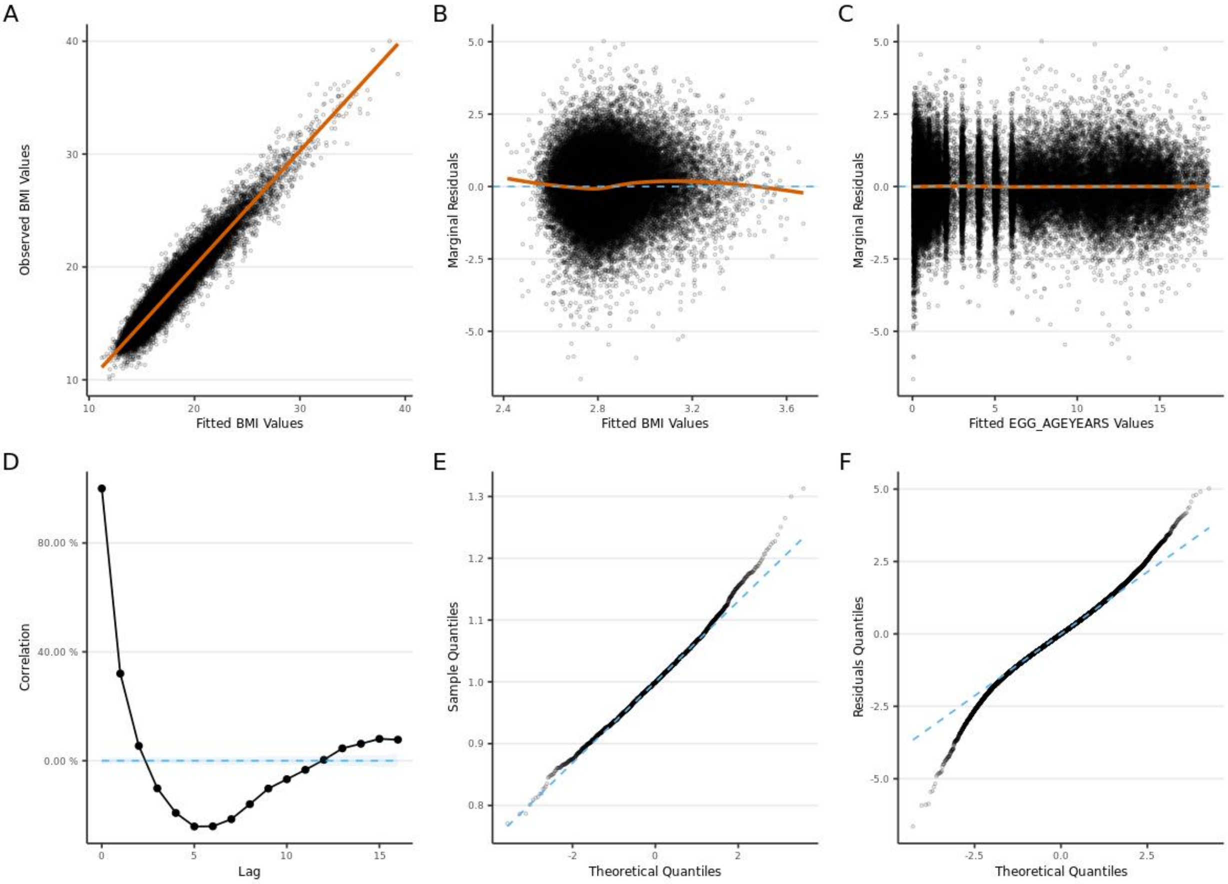
A
B
C
D
E
F
Observed BMI Values
Fitted BMI Values
Marginal Residuals
Fitted BMI Values
Marginal Residuals
Fitted EGG_AGEYEARS Values
Correlation
Lag
Sample Quantiles
Theoretical Quantiles
Residuals Quantiles
Theoretical Quantiles

6: OBE

Male

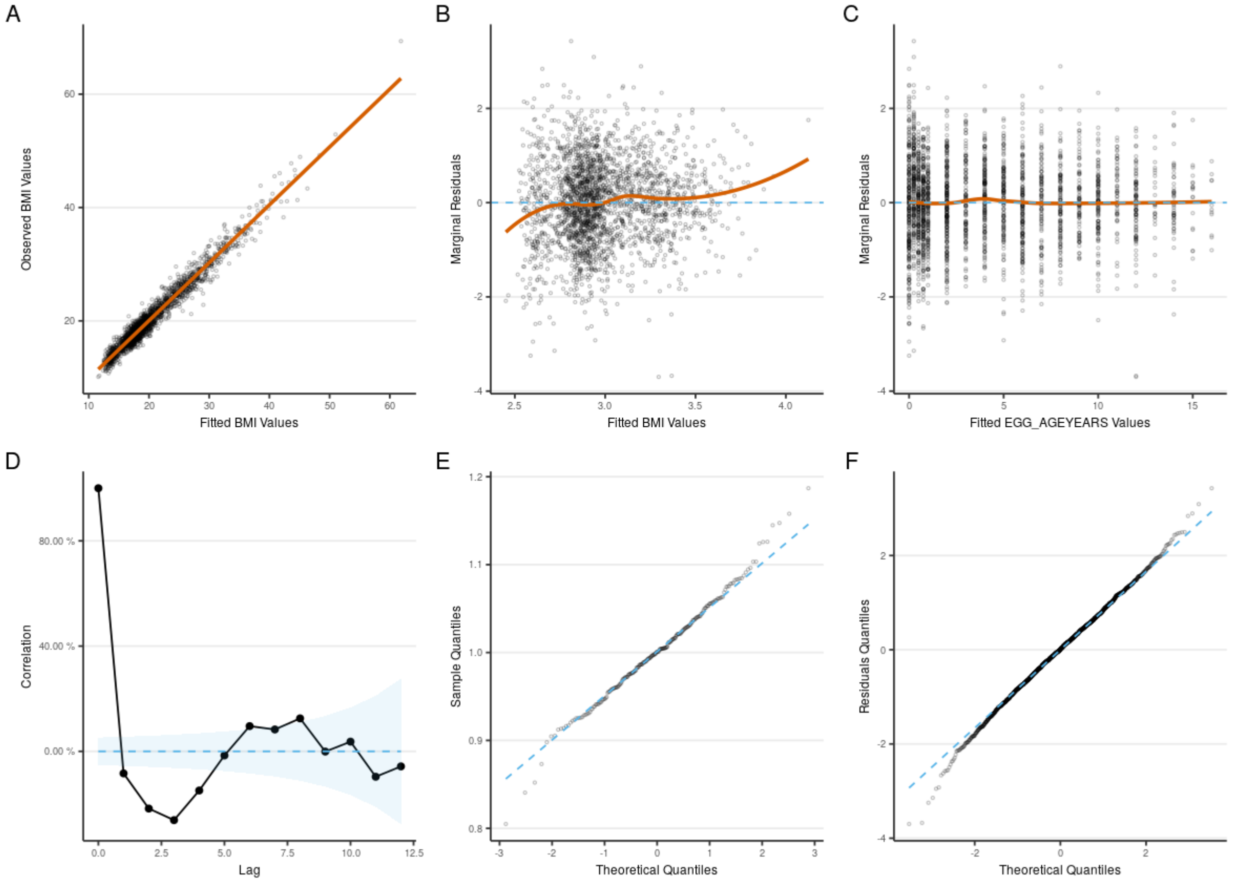
A
Observed BMI Values
Fitted BMI Values
B
Marginal Residuals
Fitted BMI Values
C
Marginal Residuals
Fitted EGG_AGEYEARS Values
D
Correlation
Lag
E
Sample Quantiles
Theoretical Quantiles
F
Residuals Quantiles
Theoretical Quantiles

Female

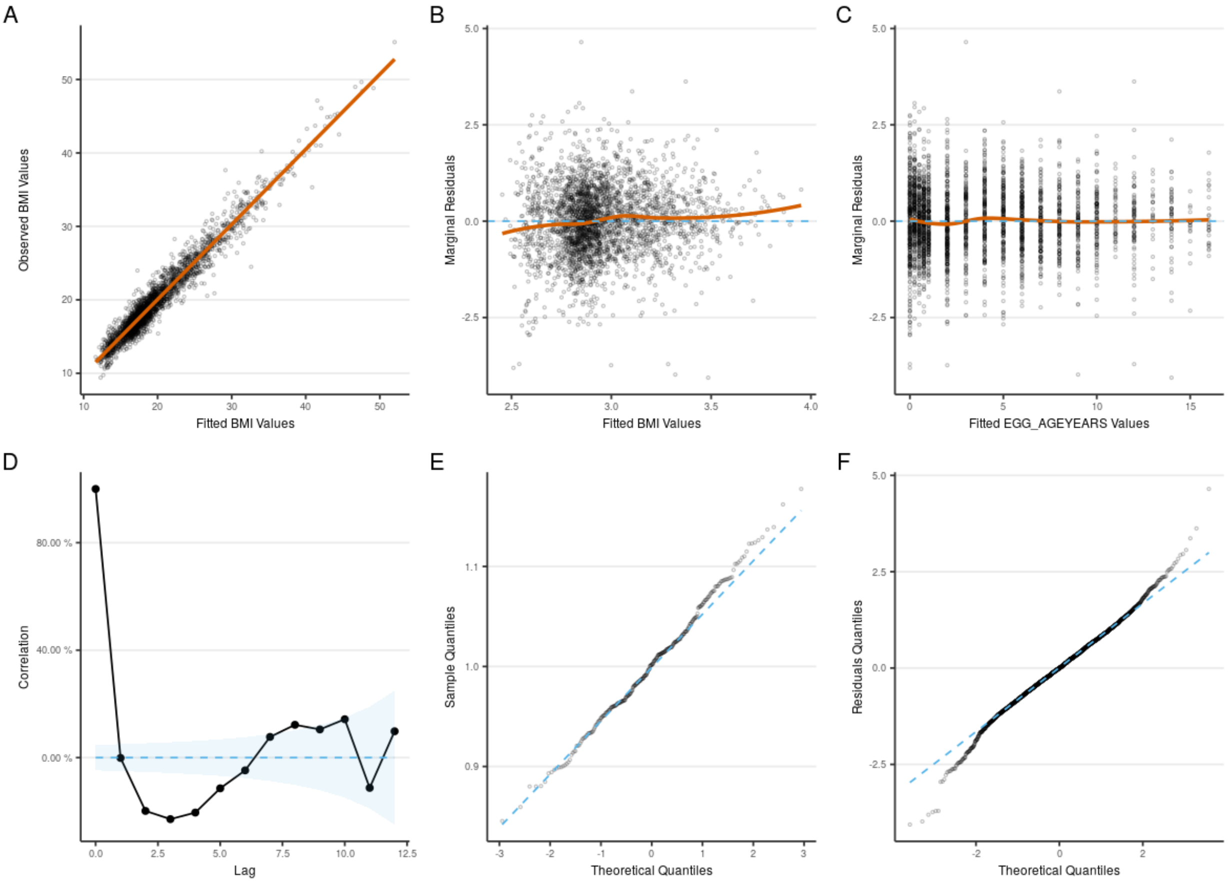
A
B
C
D
E
F
Observed BMI Values
Fitted BMI Values
Marginal Residuals
Fitted EGG_AGEYEARS Values
Correlation
Lag
Sample Quantiles
Theoretical Quantiles
Residuals Quantiles

Supplementary Figure 4: Scatter plots of the estimated phenotypes from our preferred model compared to estimated phenotypes from a model also including a continuous autoregressive correlation structure of order 1 (CAR(1)) for males and females in the ALSPAC (N= 6529 males and 6204 females) and NFBC1986 (N=2734 males and 2826 females) cohorts.

Estimated phenotypes include the age and body mass index (BMI) at the adiposity peak (AP) and adiposity rebound (AR; row 1), the slope (row two) and area under the curve (AUC; row three) across infancy (0-0.5 years), early childhood (1.5-3.5 years) and late childhood (6.5-10 years). The x-axis represents the estimated phenotypes from the preferred model and the y-axis represents the estimated phenotypes from the preferred model with correlation structure CAR(1).

ALSPAC – Males:

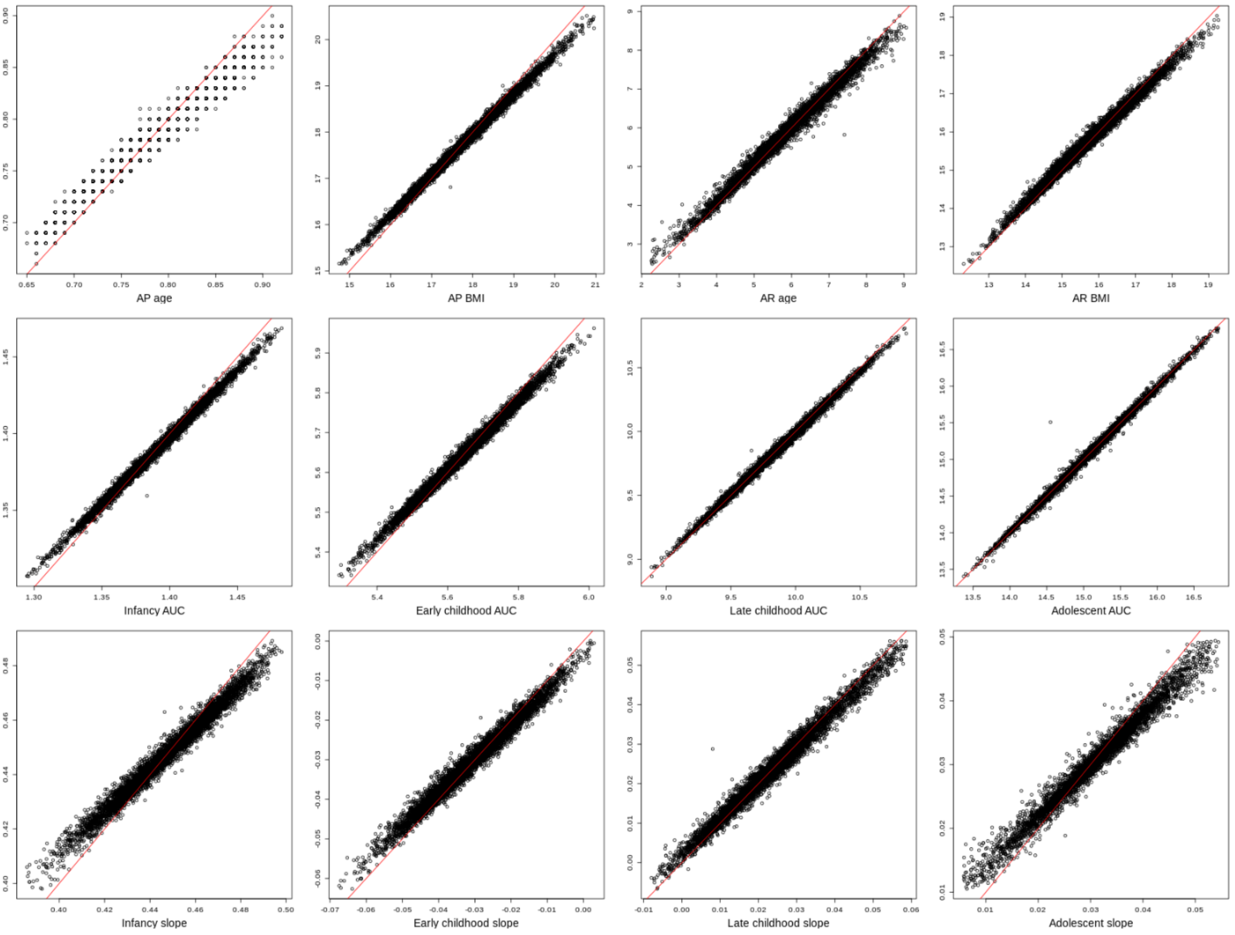

ALSPAC – Females:

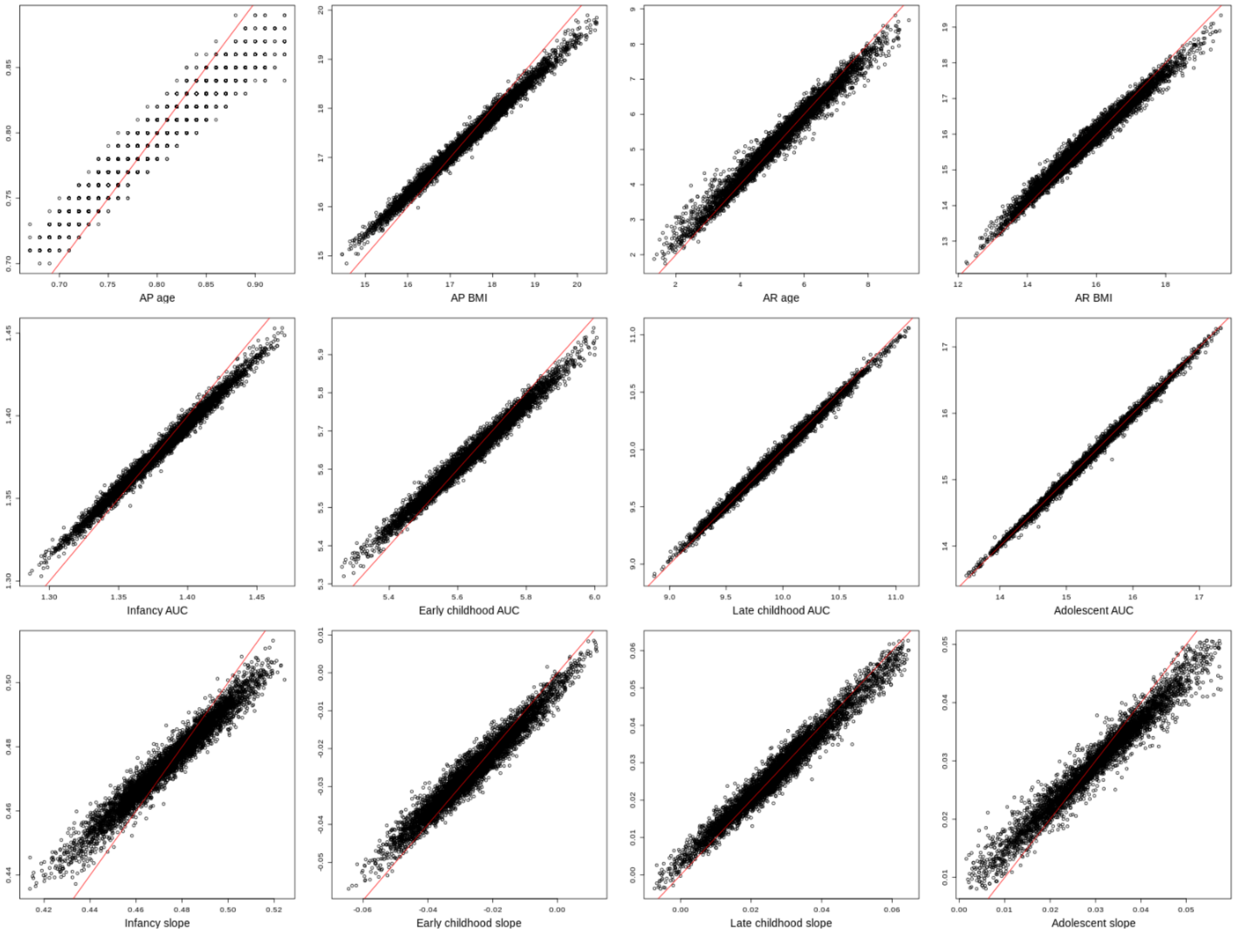

NFBC1986 - Males

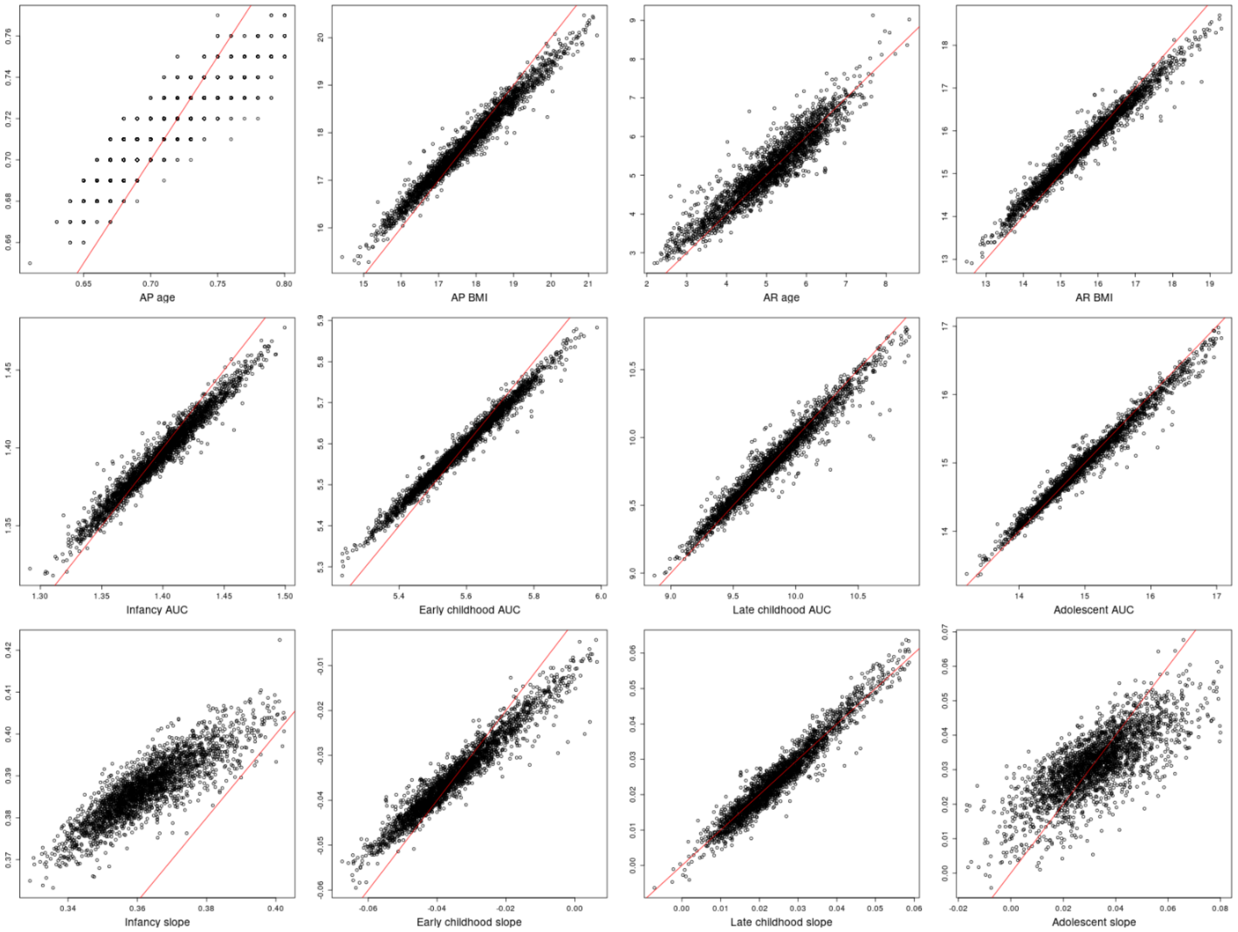

NFBC1986 – Females:

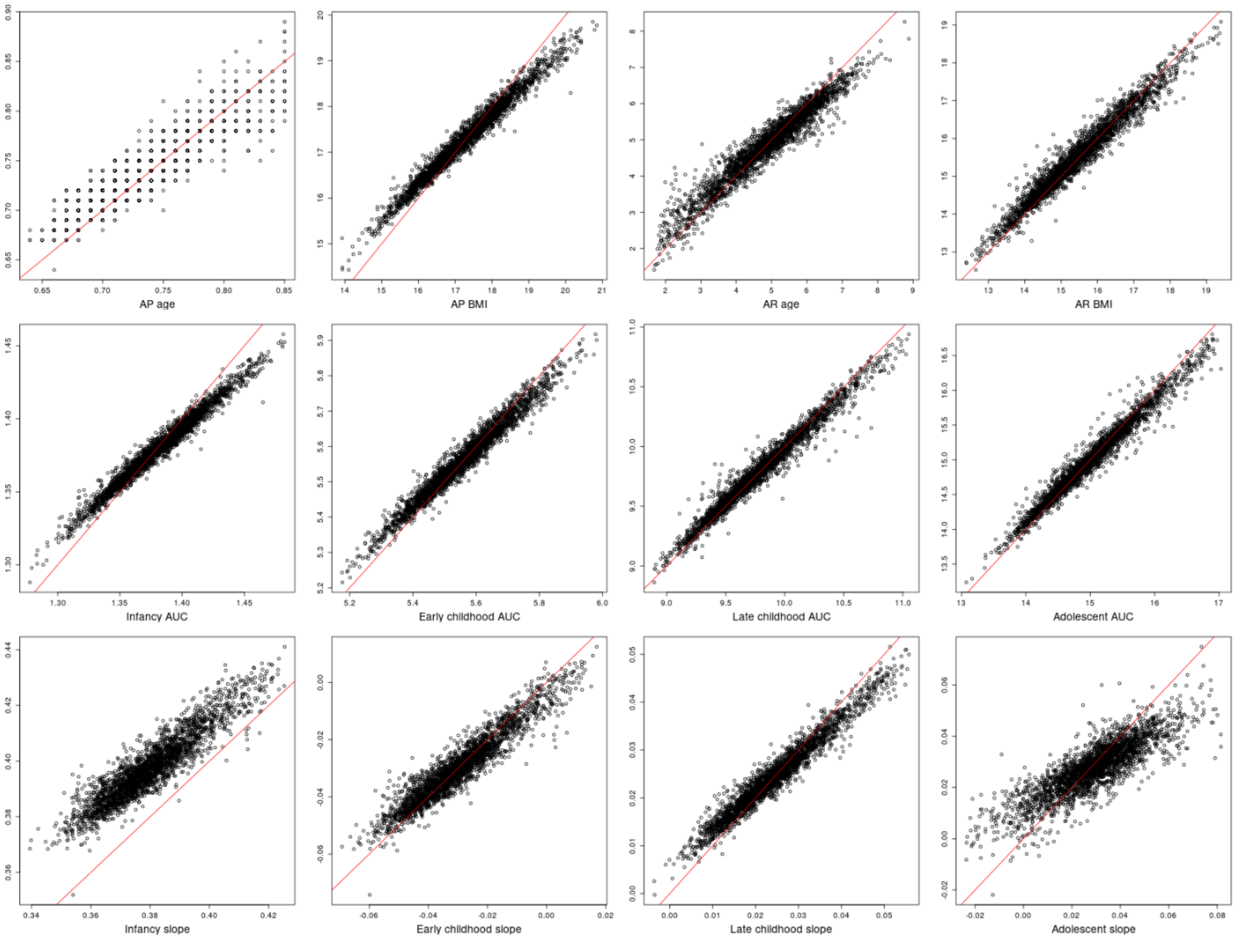

Supplementary Figure 5: Scatter plots of the estimated phenotypes from our preferred model compared to estimated phenotypes from a model including a cubic spline function both in the fixed and random effects and a continuous autoregressive correlation structure of order 1 (CAR(1)) for males and females in the ALSPAC (N= 6529 males and 6204 females) and NFBC1986 (N=2734 males and 2826 females) cohorts

Estimated phenotypes include the age and body mass index (BMI) at the adiposity peak (AP) and adiposity rebound (AR; row 1), the slope (row two) and area under the curve (AUC; row three) across infancy (0-0.5 years), early childhood (1.5-3.5 years) and late childhood (6.5-10 years). The x-axis represents the estimated phenotypes from the preferred model and the y-axis represents the estimated phenotypes from the model with cubic spline in the random effects with correlation structure CAR(1). NB: in the NFBC1986 males plots, the spline function in the comparison model includes our original knot points at 2, 8 and 12 years of age in both the fixed and random effects (whereas our preferred model had knot points at 1, 8 and 12 years).

ALSPAC – Males:

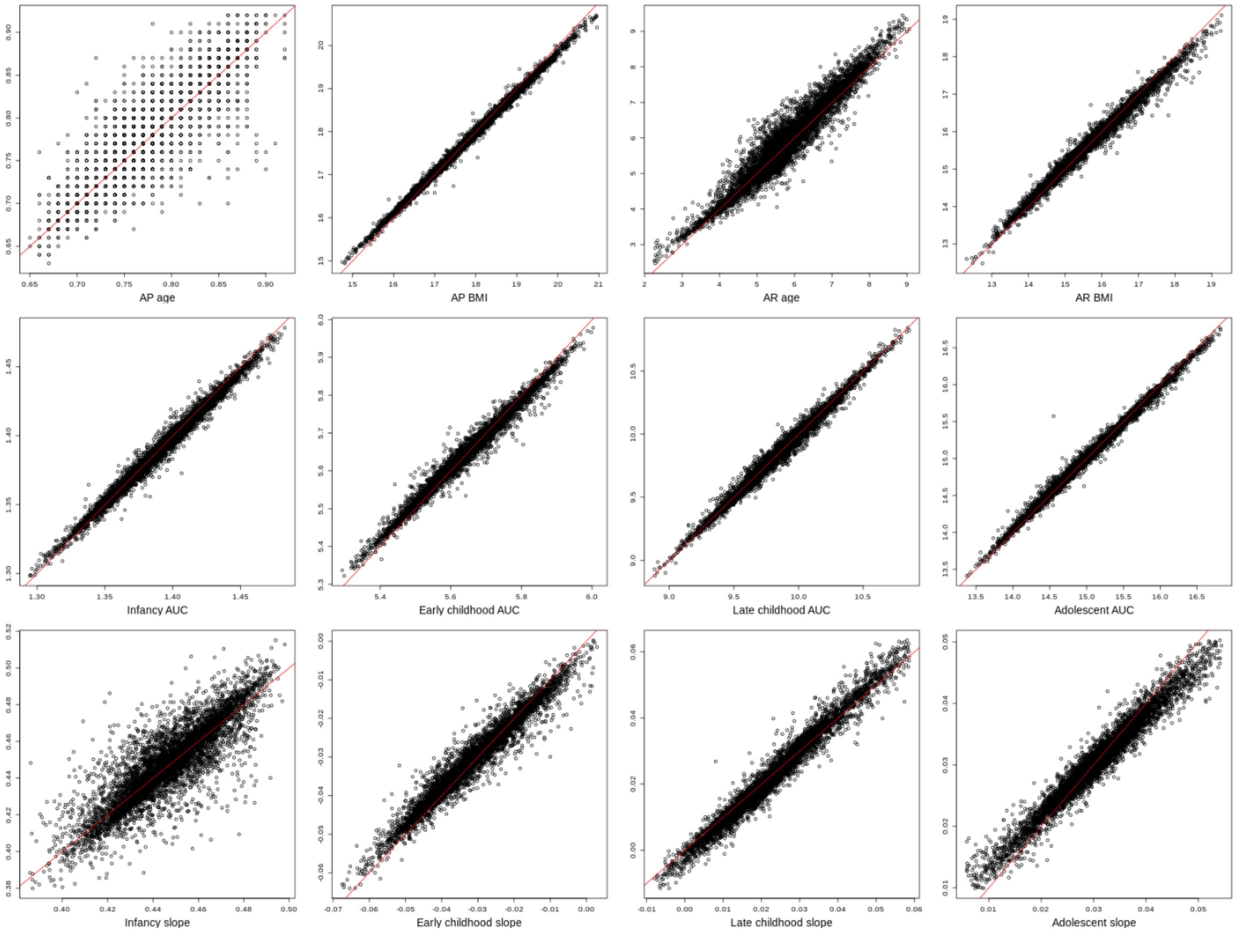

ALSPAC – Females:

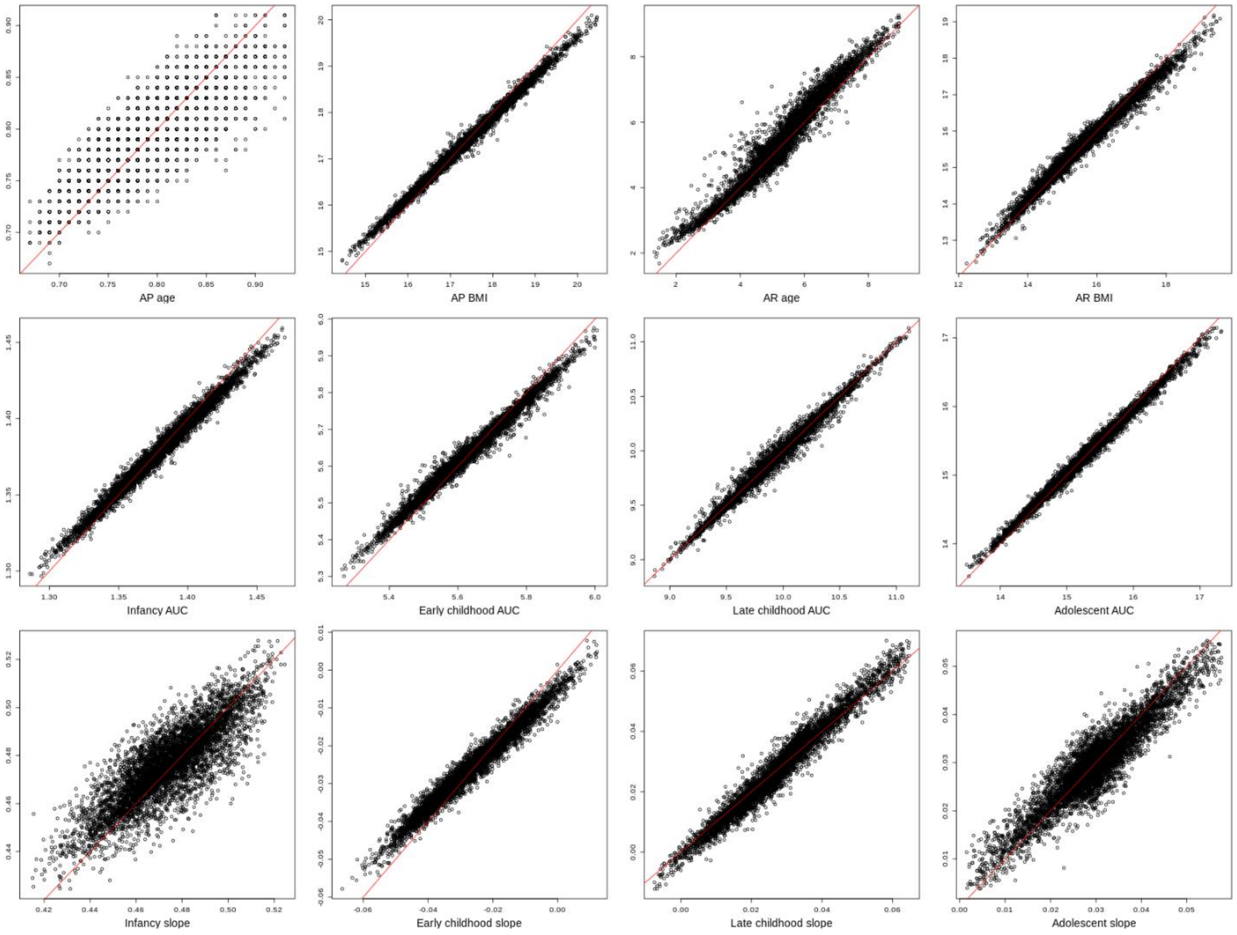

NFBC1986 – Males:

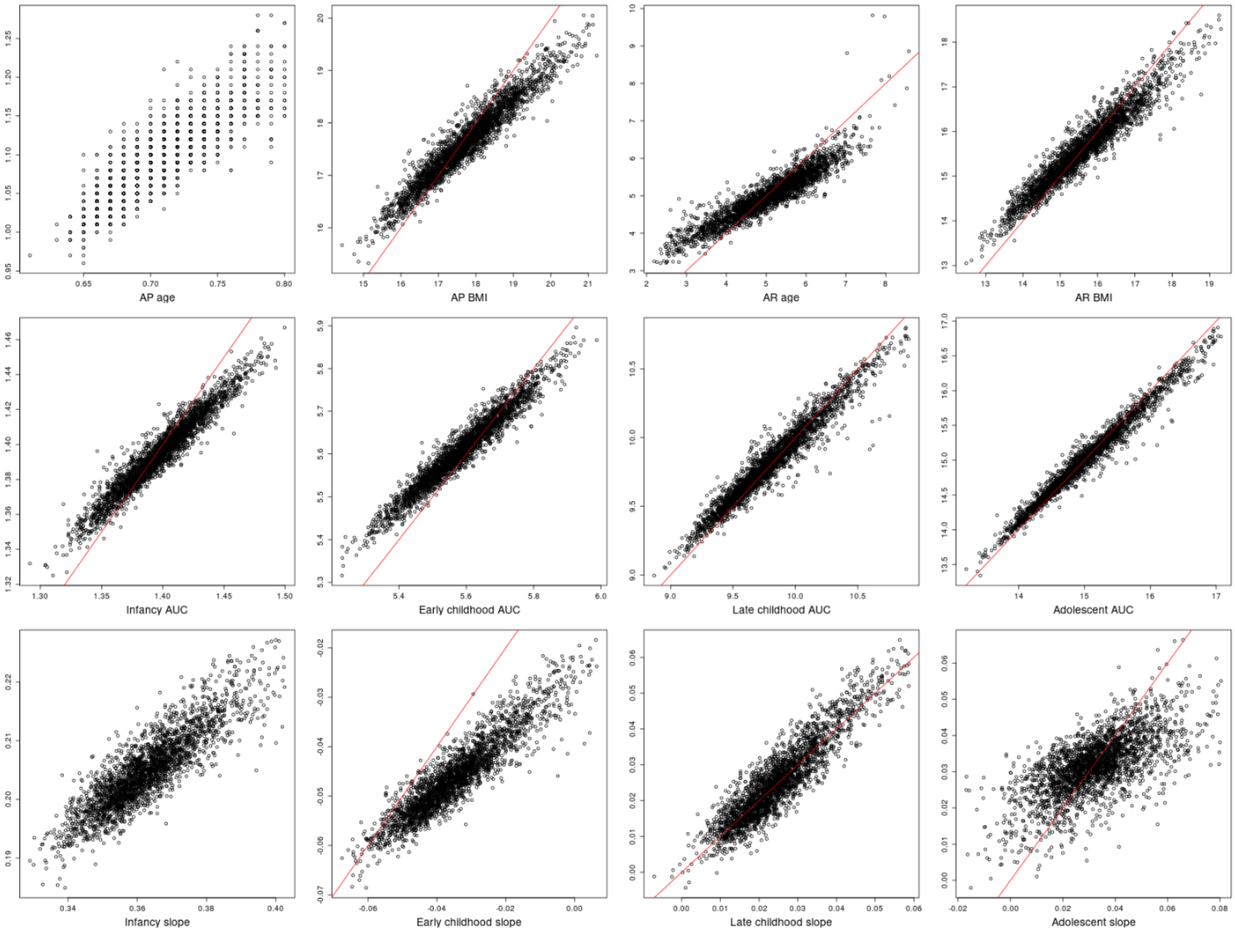

NFBC1986 – Females:

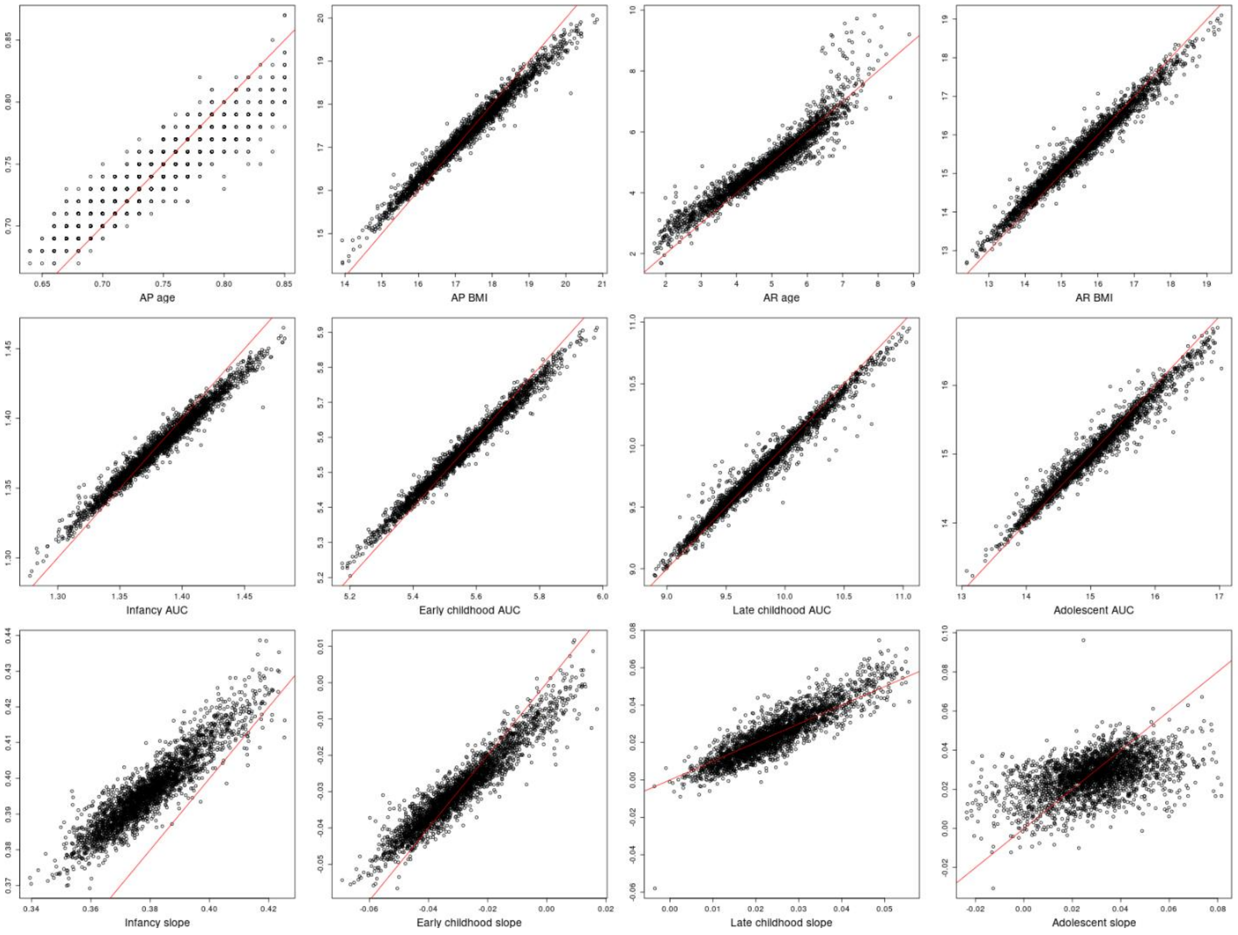

Supplementary Figure 6: Summary of the association between final BMI (between age 16-18 years) and each of the estimated phenotypes, including a random effects meta-analysis (DerSimonian-Laird estimator)

Within each cohort, both final BMI and the estimated phenotypes were standardized and the linear regression analyses were adjusted for sex. 95% confidence intervals are presented for each of the association estimates and a two-sided heterogeneity P-value is presented from the random effects meta-analysis. Sample sizes in each cohort are as follows: ALSPAC=3,663, CHOP-European: min=5,626, max=6,775, CHOP-African: min=4,903, max=5,185, NFBC1966=2,031, NFBC1986=798, OBE: min=33, max=37

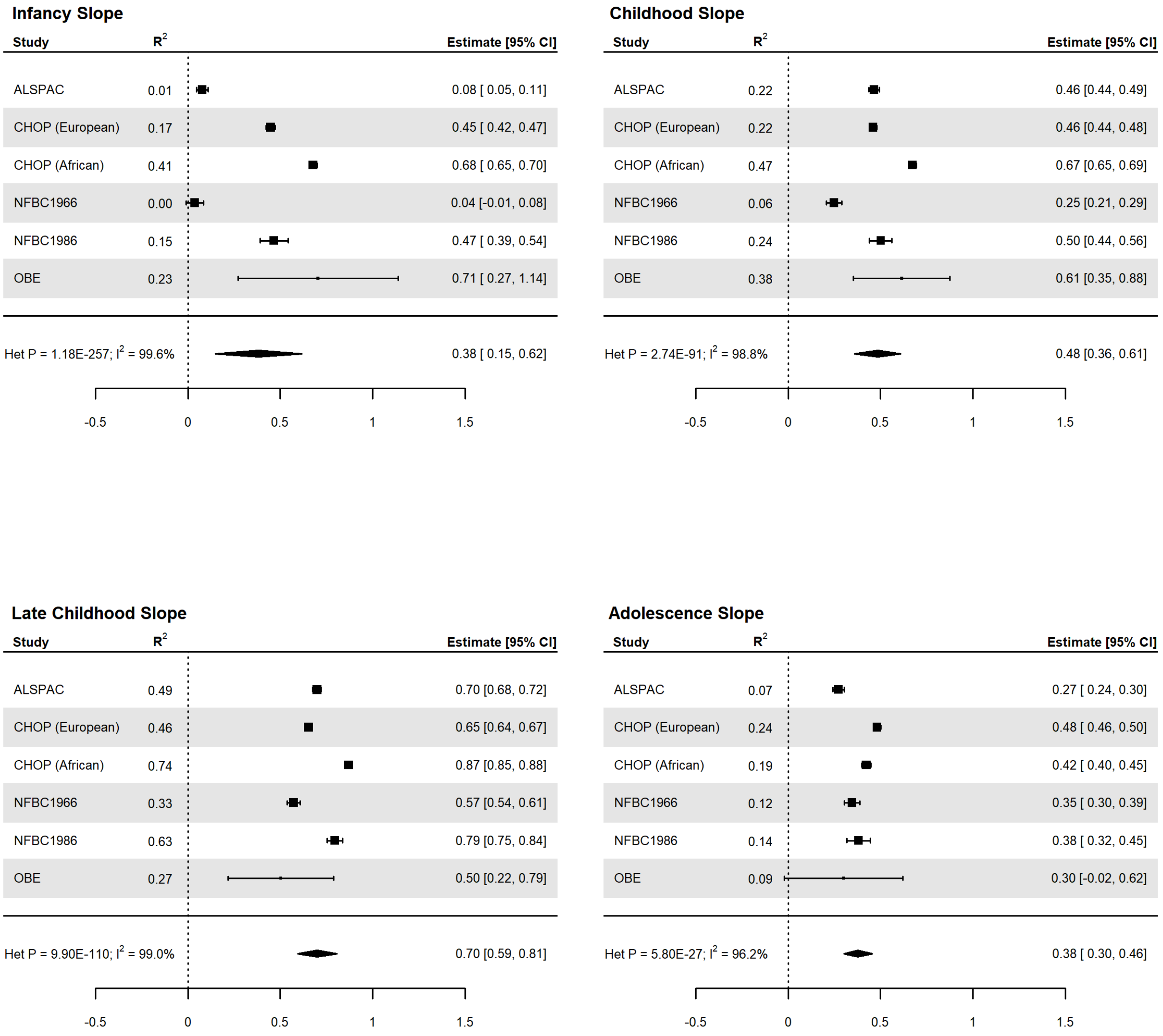

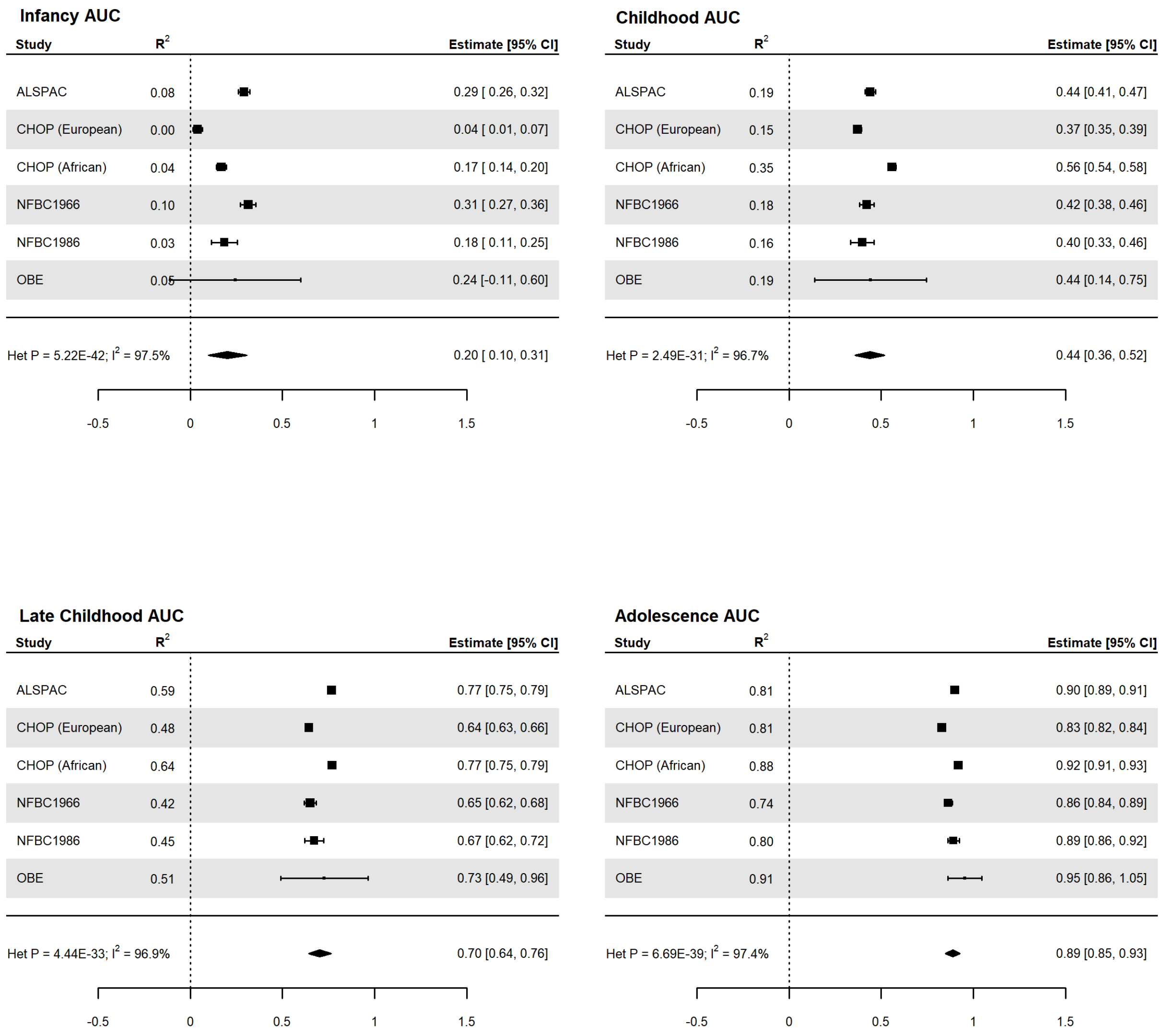
Infancy AUC
Study
R2
Estimate [95% CI]
ALSPAC 0.08 0.29 [0.26, 0.32]
CHOP (European) 0.00 0.04 [0.01, 0.07]
CHOP (African) 0.04 0.17 [0.14, 0.20]
NFBC1966 0.10 0.31 [0.27, 0.36]
NFBC1986 0.03 0.18 [0.11, 0.25]
OBE 0.05 0.24 [-0.11, 0.60]
Het P = 5.22E-42; I2 = 97.5% 0.20 [0.10, 0.31]
-0.5
0
0.5
1
1.5
Childhood AUC
Study
R2
Estimate [95% CI]
ALSPAC 0.19 0.44 [0.41, 0.47]
CHOP (European) 0.15 0.37 [0.35, 0.39]
CHOP (African) 0.35 0.56 [0.54, 0.58]
NFBC1966 0.18 0.42 [0.38, 0.46]
NFBC1986 0.16 0.40 [0.33, 0.46]
OBE 0.19 0.44 [0.14, 0.75]
Het P = 2.49E-31; I2 = 96.7% 0.44 [0.36, 0.52]
-0.5
0
0.5
1
1.5
Late Childhood AUC
Study
R2
Estimate [95% CI]
ALSPAC 0.59 0.77 [0.75, 0.79]
CHOP (European) 0.48 0.64 [0.63, 0.66]
CHOP (African) 0.64 0.77 [0.75, 0.79]
NFBC1966 0.42 0.65 [0.62, 0.68]
NFBC1986 0.45 0.67 [0.62, 0.72]
OBE 0.51 0.73 [0.49, 0.96]
Het P = 4.44E-33; I2 = 96.9% 0.70 [0.64, 0.76]
-0.5
0
0.5
1
1.5
Adolescence AUC
Study
R2
Estimate [95% CI]
ALSPAC 0.81 0.90 [0.89, 0.91]
CHOP (European) 0.81 0.83 [0.82, 0.84]
CHOP (African) 0.88 0.92 [0.91, 0.93]
NFBC1966 0.74 0.86 [0.84, 0.89]
NFBC1986 0.80 0.89 [0.86, 0.92]
OBE 0.91 0.95 [0.86, 1.05]
Het P = 6.69E-39; I2 = 97.4% 0.89 [0.85, 0.93]
-0.5
0
0.5
1
1.5

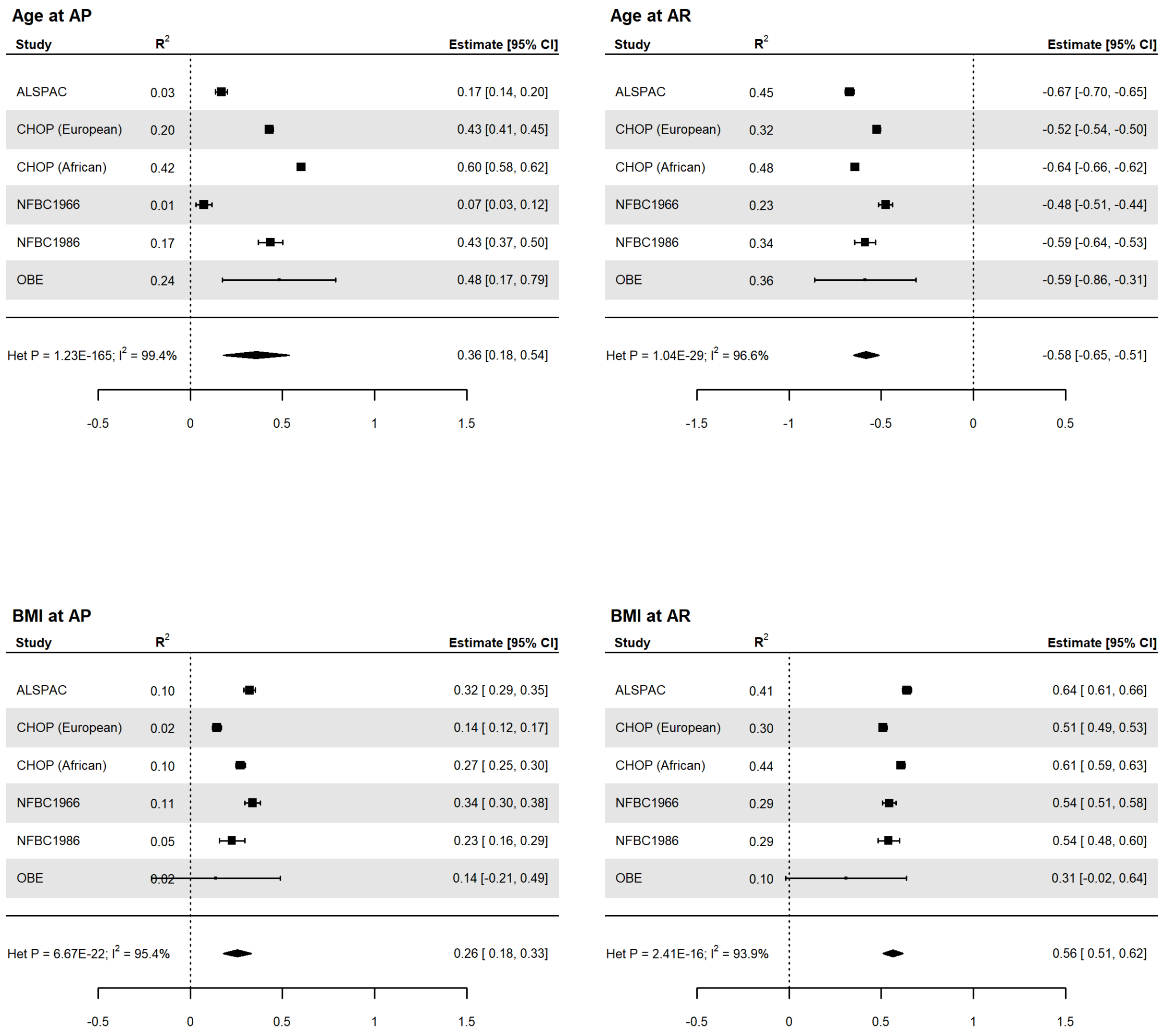
Age at AP
Study
R^2
Estimate [95% CI]
ALSPAC 0.03 0.17 [0.14, 0.20]
CHOP (European) 0.20 0.43 [0.41, 0.45]
CHOP (African) 0.42 0.60 [0.58, 0.62]
NFBC1966 0.01 0.07 [0.03, 0.12]
NFBC1986 0.17 0.43 [0.37, 0.50]
OBE 0.24 0.48 [0.17, 0.79]
Het P = 1.23E-165; I^2 = 99.4% 0.36 [0.18, 0.54]
-0.5 0 0.5 1 1.5
Age at AR
Study
R^2
Estimate [95% CI]
ALSPAC 0.45 -0.67 [-0.70, -0.65]
CHOP (European) 0.32 -0.52 [-0.54, -0.50]
CHOP (African) 0.48 -0.64 [-0.66, -0.62]
NFBC1966 0.23 -0.48 [-0.51, -0.44]
NFBC1986 0.34 -0.59 [-0.64, -0.53]
OBE 0.36 -0.59 [-0.86, -0.31]
Het P = 1.04E-29; I^2 = 96.6% -0.58 [-0.65, -0.51]
-1.5 -1 -0.5 0 0.5
BMI at AP
Study
R^2
Estimate [95% CI]
ALSPAC 0.10 0.32 [0.29, 0.35]
CHOP (European) 0.02 0.14 [0.12, 0.17]
CHOP (African) 0.10 0.27 [0.25, 0.30]
NFBC1966 0.11 0.34 [0.30, 0.38]
NFBC1986 0.05 0.23 [0.16, 0.29]
OBE 0.02 0.14 [-0.21, 0.49]
Het P = 6.67E-22; I^2 = 95.4% 0.26 [0.18, 0.33]
-0.5 0 0.5 1 1.5
BMI at AR
Study
R^2
Estimate [95% CI]
ALSPAC 0.41 0.64 [0.61, 0.66]
CHOP (European) 0.30 0.51 [0.49, 0.53]
CHOP (African) 0.44 0.61 [0.59, 0.63]
NFBC1966 0.29 0.54 [0.51, 0.58]
NFBC1986 0.29 0.54 [0.48, 0.60]
OBE 0.10 0.31 [-0.02, 0.64]
Het P = 2.41E-16; I^2 = 93.9% 0.56 [0.51, 0.62]
-0.5 0 0.5 1 1.5

Supplementary Figure 7: Quantile-quantile (QQ) plots of the heterogeneity tests in the European inverse-variance-weighted fixed-effects meta-analyses for each of the estimated phenotypes

Estimated phenotypes include the age and body mass index (BMI) at the adiposity peak (AP) and adiposity rebound (AR; row 1), the slope (row two) and area under the curve (AUC; row three) across infancy (0-0.5 years), early childhood (1.5-3.5 years) and late childhood (6.5-10 years).

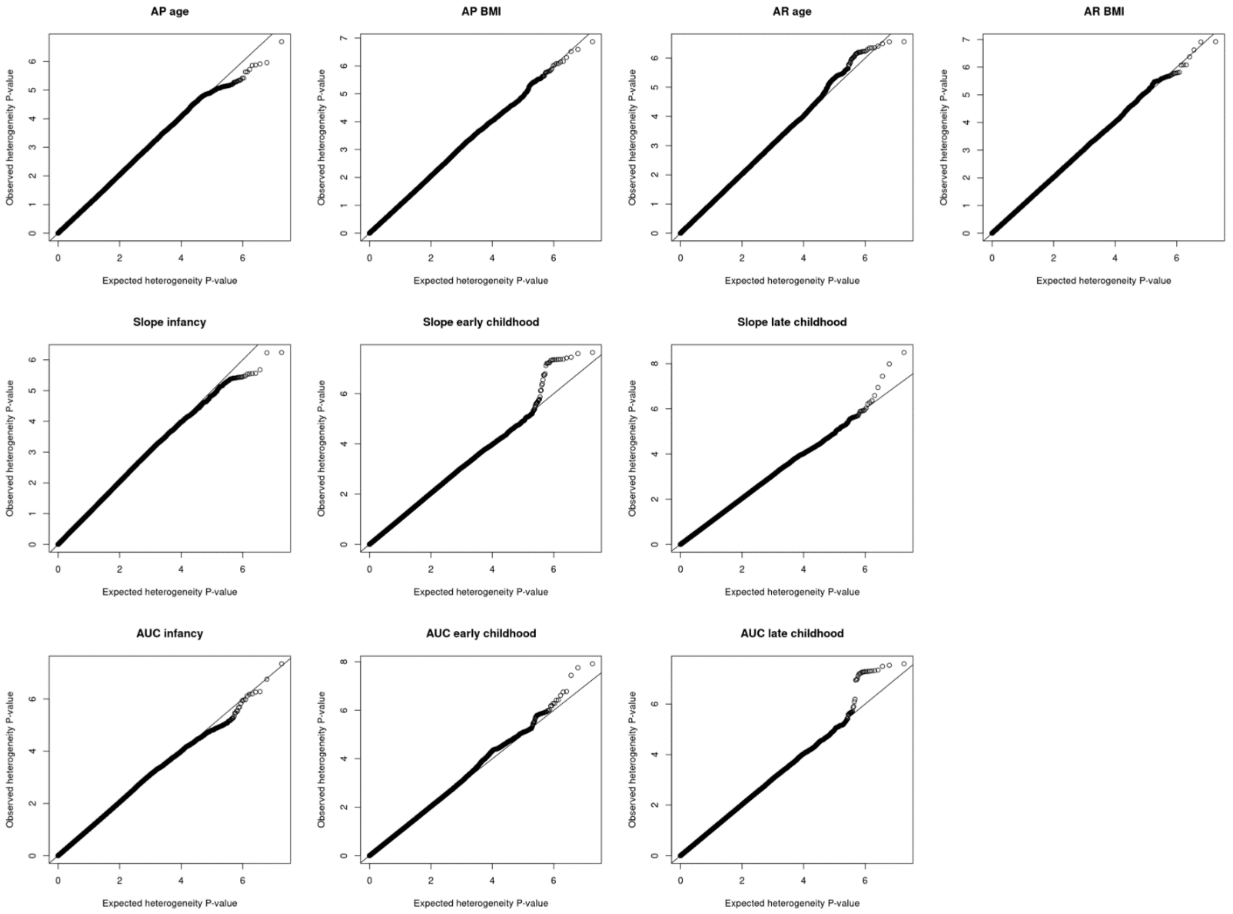

Supplementary Figure 8: Quantile-quantile (QQ) plots of the heterogeneity test in the European inverse-variance-weighted fixed-effects meta-analyses for each of the estimated phenotypes, excluding the OBE cohort.

Estimated phenotypes include the age and body mass index (BMI) at the adiposity peak (AP) and adiposity rebound (AR; row 1), the slope (row two) and area under the curve (AUC; row three) across infancy (0-0.5 years), early childhood (1.5-3.5 years), late childhood (6.5-10 years) and adolescence (12-17 years).

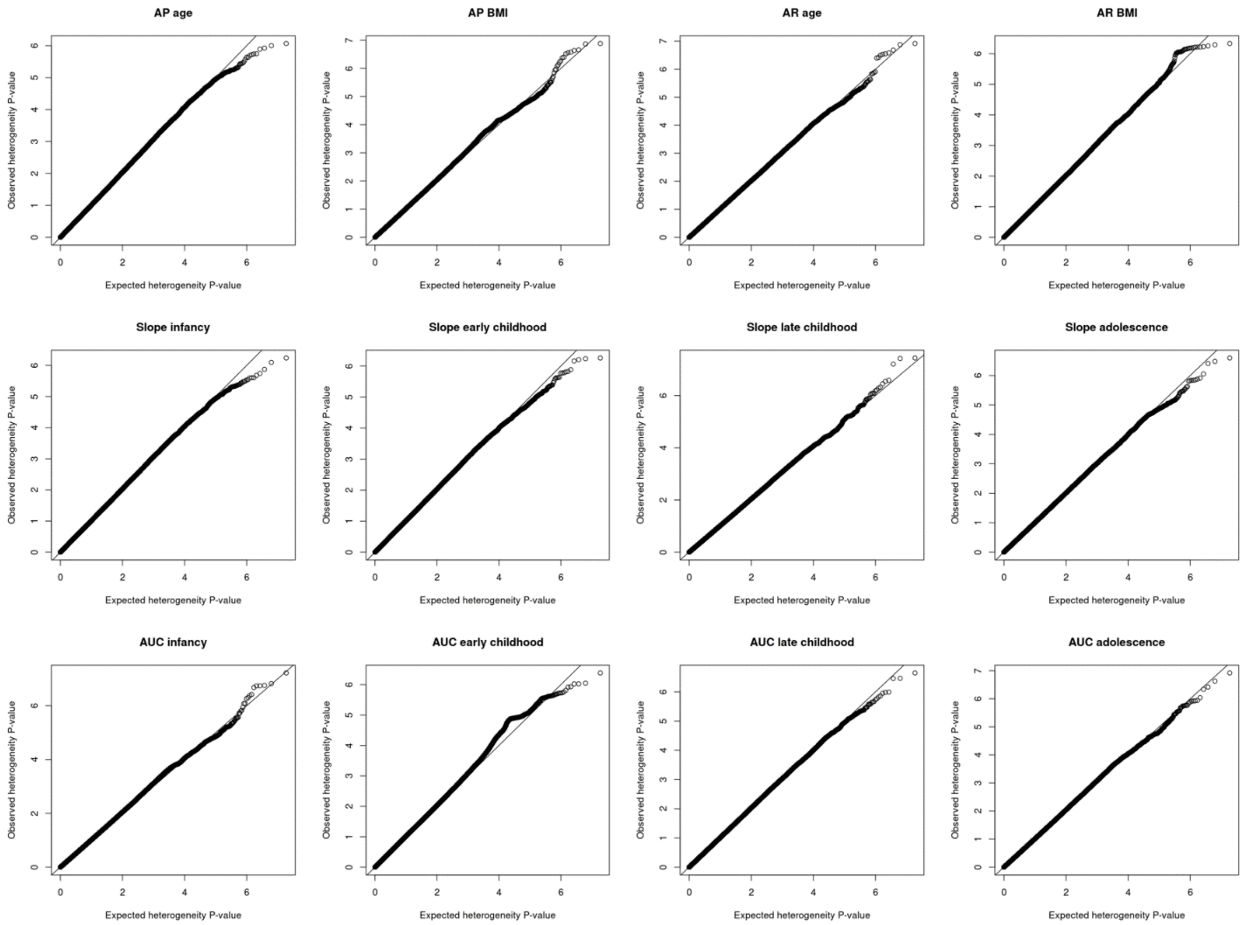

Supplementary Figure 9: Manhattan plots of the meta-analyses without OBE for the phenotypes across infancy (0-0.5 years), early childhood (1.5-3.5 years), late childhood (6.5-10 years) and adolescence (12-17 years)

The two-sided association P-value on the $-\log_{10}$ scale obtained from the inverse-variance-weighted fixed-effects meta-analysis for each of the SNPs (y-axis) was plotted against the genomic position (NCBI Build 37; x-axis). Loci are labelled with their nearest gene annotated by LocusZoom. The red line corresponds to the genome-wide significance level of $P<5x10^{-8}$, which accounts for multiple testing.

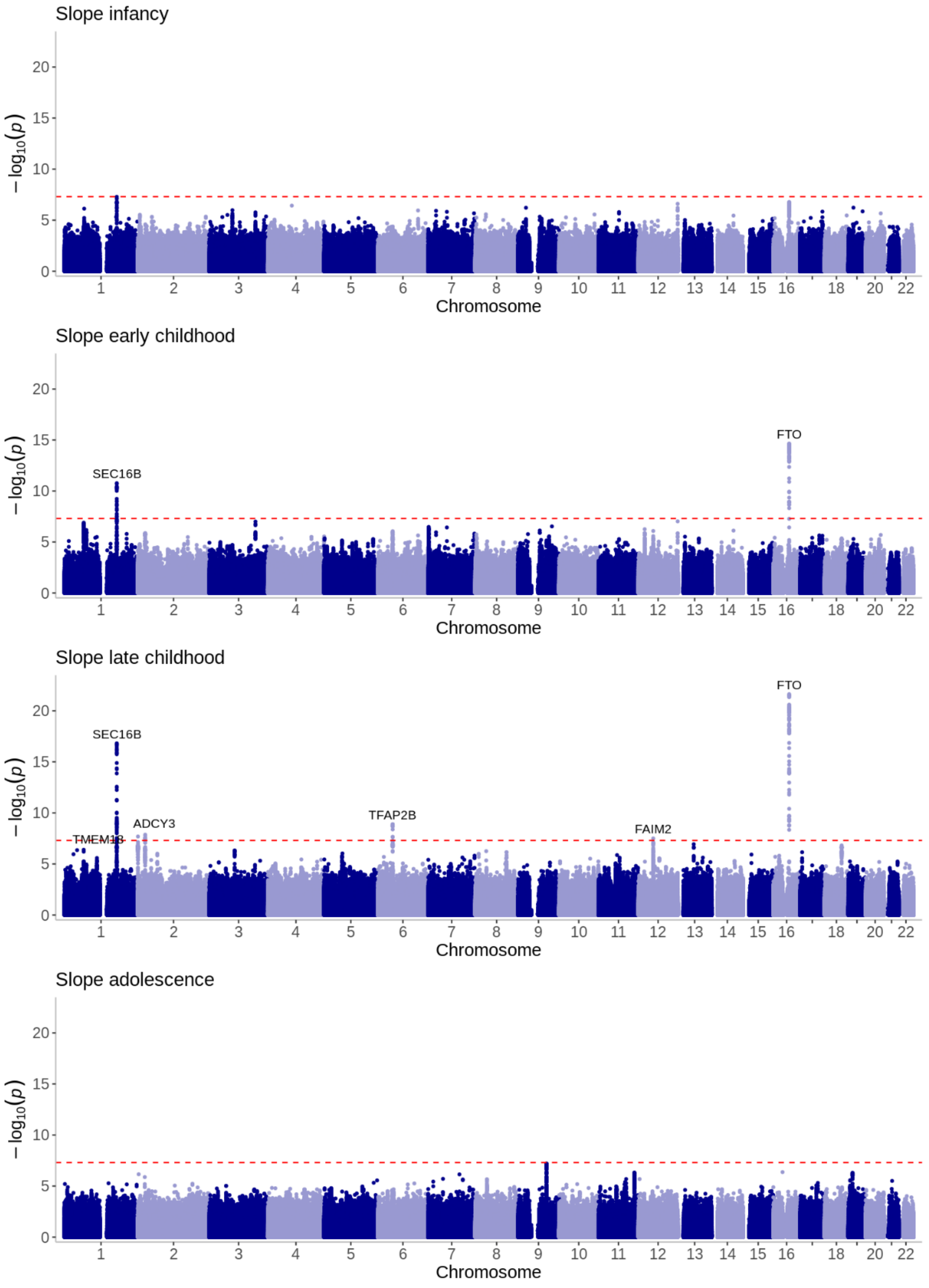

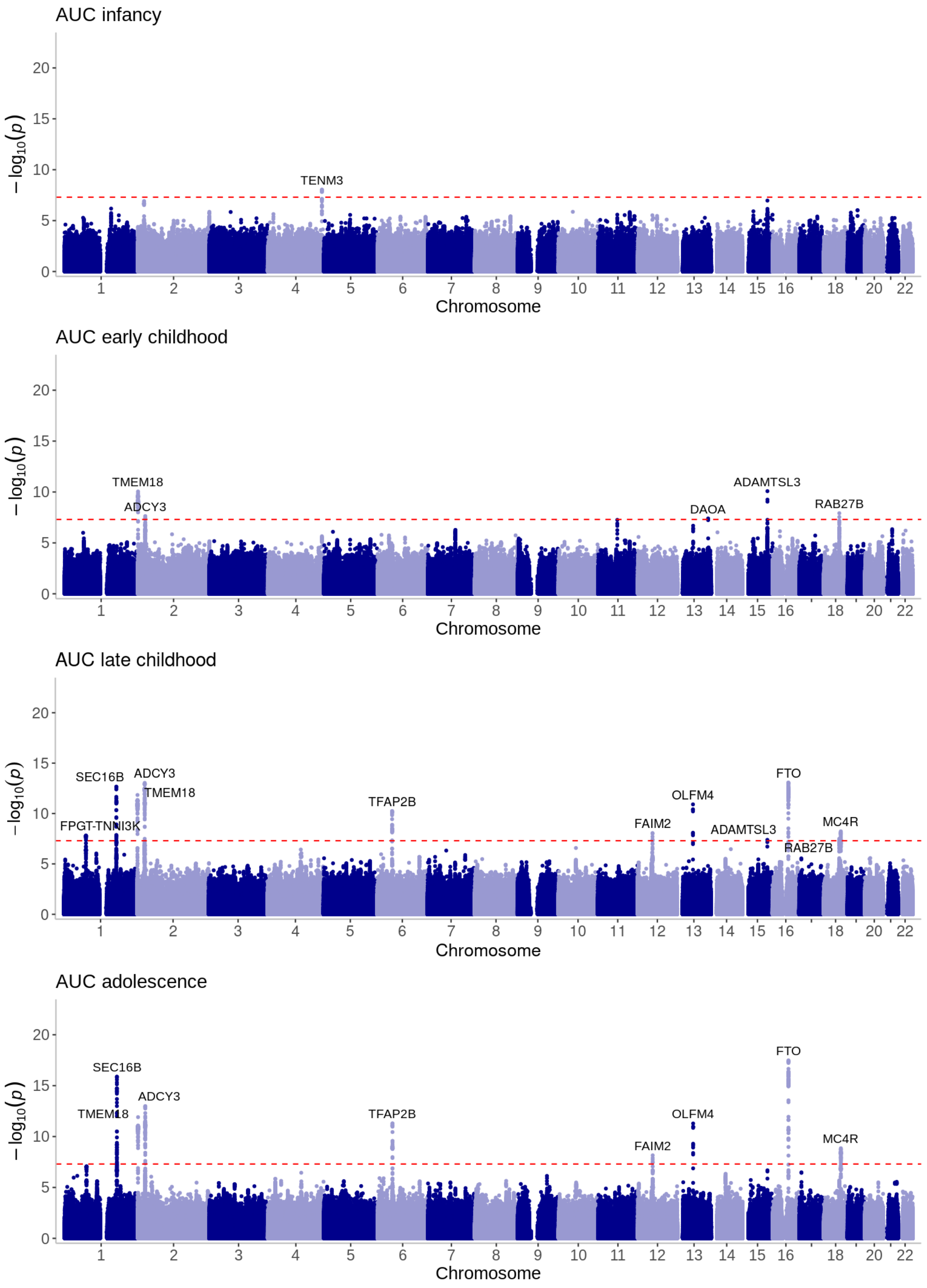
AUC infancy
TENM3
AUC early childhood
TMEM18
ADCY3
DAOA
ADAMTSL3
RAB27B
AUC late childhood
SEC16B
ADCY3
TMEM18
FPGT-TNNI3K
TFAP2B
FAIM2
OLFM4
ADAMTSL3
FTO
RAB27B
MC4R
AUC adolescence
SEC16B
TMEM18
ADCY3
TFAP2B
FAIM2
OLFM4
FTO
MC4R
$-\log_{10}(p)$
Chromosome

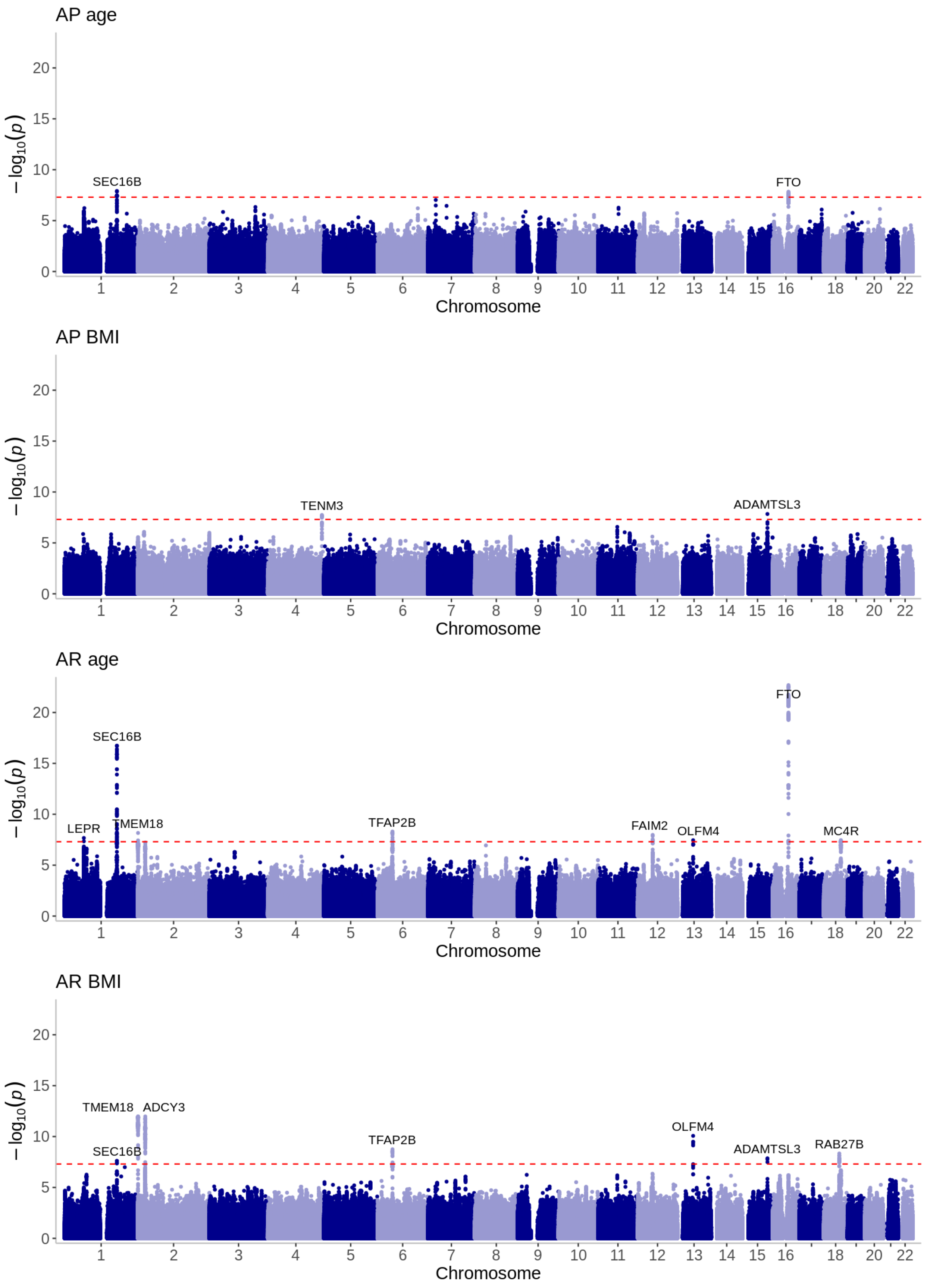
AP age
SEC16B
FTO
AP BMI
TENM3
ADAMTSL3
AR age
FTO
SEC16B
LEPR
TMEM18
TFAP2B
FAIM2
OLFM4
MC4R
AR BMI
TMEM18
ADCY3
SEC16B
TFAP2B
OLFM4
ADAMTSL3
RAB27B
$-\log_{10}(p)$
Chromosome
1 2 3 4 5 6 7 8 9 10 11 12 13 14 15 16 18 20 22
0 5 10 15 20

Supplementary Figure 10: LocusZoom regional plot of the association between adolescent slope and the FAM120AOS locus. The two-sided association P-value on the $-\log_{10}$ scale obtained from the inverse-variance-weighted fixed-effects meta-analysis for each of the SNPs (y-axis) was plotted against the genomic position (NCBI Build 37; x-axis) for all SNPs within the region.

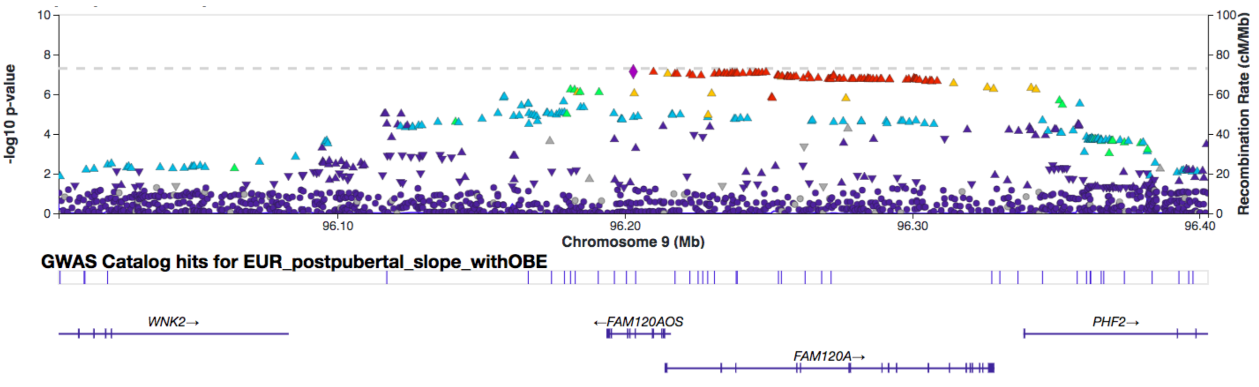

Supplementary Notes

<u>Supplementary Note 1:</u> Membership of the Early Growth Genetics (EGG) Consortium
Full list of members (May 2024), in alphabetical order.

Linda S Adair[23], Emma Ahlqvist[24], Tarunveer S Ahluwalia[25,26,27], Sonia Anand[28,29,30], Izzuddin Aris[31], Mustafa Atalay[32], Shiu Lun Ryan Au Yeung[33], Jennifer Baker[34], Zhanna Balkhiiarova[35], Robin N Beaumont[36], Jeffrey J Beck[37,38], Jose Ramon Bilbao[39,40,41], Thomas Bond[42,43,44,45], Klaus Bønnelykke[25], Dorret I Boomsma[46,47,48], Jonathan P Bradfield[49,50], Winter Bruner[51], Mariona Bustamante[52,53,54], Yee-Ming Chan[55,56,57], Aminata H Cissé[36], Gemma Clayton[45], Lachlan Coin[58], Cyrus Cooper[59], William E Copeland[60], Diana L Cousminer[61,62,63], Alexessander Couto-Alves[64], John A Curtin[65], Adnan Custovic[66], Felix R Day[67], George Dedoussis[68], Jessica Dennis[69], Maneka De Silva[70], Anders Eliasen[25], Paul Elliott[42], Aino-Maija Eloranta[71], Johan Eriksson[72,73,74], David Evans[58,44,45], João Fadista[75], Bjarke Feenstra[76], Janine Felix[77,78], Joshua Fisher[79], Christopher Flatley[80], Tim Frayling[36], Rachel M Freathy[36], Romy Gaillard[81], Nina Rica Wium Geiker[82], Frank Geller[76], Joseph T Glessner[83,84,85], Struan FA Grant[51,86,84,85,49], Niels A Grarup[87], Leif Groop[88,24], Monica Guxens[89,53,90,91], Dexter Hadley[92], Hakon Hakonarson[93], Torben Hansen[87], Andrew T Hattersley[36], M Geoffrey Hayes[94,95,96], Johannes Hebebrand[97,98], Sami Heikkinen[32], Joachim Heinrich[99], Anni Heiskala[100], Øyvind Helgeland[101,102,103], Tine Brink Henrisksen[104], Anke Hinney[97,98], Joel N Hirschhorn[56], Marie-France Hivert[31,105,106], Berthold Hocher[107,108,109], John W Holloway[110], Jens-Christian Holm[111], Momoko Horikoshi[112], Jouke-Jan Hottenga[113,114,115], Yun Huang[87], Alice E Hughes[36], Elina Hyppönen[116,117], Bo Jacobsson[118,119], Vincent Jaddoe[77,78], Annika Jaitner[36], Marjo-Riitta Järvelin[120,121,122,123,124], Stefan Johansson[102,125], Marika A Kaakinen[126,127,128], Heidi J Kalkwarf[129], Irfahan Kassam[130], Antje Körner[131,132,133], Sailesh Kotecha[134], Zoltán Kutalik[135,136,137], Timo A Lakka[138], Amel Lamri[139,29], Joan M Lappe[140], Deborah A Lawlor[45,48], Terho Lehtimäki[141,142,143], Alexandra M Lewin[144], Brandon Lim[36], Cecilia M Lindgren[145,146], Virpi Lindi[32], Allan Linneberg[34,147], Jun Liu[148], William L Lowe Jr[94], Leo-Pekka Lyytikäinen[141,142,143], Ronald CW Ma[149,150,151], Reedik Mägi[152], Per Magnus[153], Mark I McCarthy[154,155], Nina S McCarthy[156], Mads Melbye[157,158,159,160], Gunn-Helen Moen[58,161,160,48], Karen L Mohlke[162], Dennis Mook-Kanamori[163,164], Andrew P Morris[165], Ehsan Motazedi[166], Jeffrey C Murray[167], Ronny Myhre[168], Juha Mykkänen[169,170], Bhargav Ganesh Naveen[36], Harri Niinikoski[169,170,171], Pal Rasmus Njølstad[172,173], Ellen Aagaard Nohr[174], Ioanna Ntalla[175], Sharon E Oberfield[176], Emily Oken[31], Ken Ong[67,177], Katja Pahkala[169,170,178], Oluf Pedersen[87], Craig E Pennell[179,180], John RB Perry[67], Triinu Peters[97], Roseann E Peterson[181], Niina Pitkänen[169], Christine Power[182], Rashmi B Prasad[24,183], Inga Prokopenko[35,184,185], Olli T Raitakari[169,170,186], Rebecca M Reynolds[187], Rebecca C Richmond[188,48], Alina Rodriguez[189,190,] Justiina Ronkainen[100], Rany Salem[191,192,193,56], Seang Mei Saw[194,195], William Schierding[196], Theresia M Schnurr[87], Sylvain Sebert[197], Angela Simpson[65], Line Skotte[75], Pol Sole-Navais[118], Thorkild IA Sørensen[87], David Stacey[116], Marie Standl[198], Eric AP Steegers[199], Sara Elizabeth Stinson[87], Beate St Pourcain[200,188], David Strachan[201], Jordi Sunyer[52,53,54,202], Yik Ying Teo[203,204], Elisabeth Thiering[198,205], Nicholas J Timpson[45], Jessica Tyrrell[36,206], Andre G Uitterlinden[207], Cornelia van Dujin[208,209], Marc Vaudel[210,119], Martine Vrijheid[52,53,54], Tanja Vrijkotte[166], Carol A Wang[179,180], Nicole M Warrington[58,44,160], H-Erich Wichmann[211,212,213], Elisabeth Widén[241], Gonneke Willemsen[215], James F Wilson[216], Xiaoping Wu[76], Eleftheria Zeggini[217,218], Babette S Zemel[219], Ge Zhang[220,221,222,223], Jia Zhu[55]

[23]Department of Nutrition, University of North Carolina, Chapel Hill, NC, USA, [24]Department of Clinical Sciences, Diabetes and Endocrinology, Lund University Diabetes Centre, Malmö, Sweden, [25]COPSAC, Copenhagen Prospective Studies on Asthma in Childhood, Herlev and Gentofte Hospital, University of Copenhagen, Copenhagen, Denmark, [26]Steno Diabetes Center Copenhagen, Herlev, Denmark, [27]The Bioinformatics Center, Department of Biology, University of Copenhagen, Copenhagen, Denmark, [28]Department of Medicine, McMaster University, Hamilton, Ontario, Canada., [29]Population Health Research Institute, Hamilton Health Sciences and McMaster University, Hamilton, Ontario, Canada, [30]Department of Health Research Methods, Evidence, and Impact, McMaster University, Hamilton, Ontario, Canada., [31]Department of Population Medicine, Harvard Pilgrim Health Care Institute, Harvard Medical School, Boston, USA, [32]Institute of Biomedicine, School of Medicine, University of Eastern Finland, Kuopio Campus, Finland, [33]School of Public Health, The University of Hong Kong, Hong Kong, China, [34]Center for Clinical Research

and Prevention, Bispebjerg and Frederiksberg Hospital, Copenhagen, Denmark, [35]Department of Clinical & Experimental Medicine, University of Surrey, Guildford, UK, [36]Department of Biomedical and Clinical Sciences, Faculty of Health and Life Sciences, University of Exeter, Exeter, UK, [37]Avera Institute for Human Genetics, Avera McKennan Hospital and University Health Center, Sioux Falls, South Dakota, USA, [38]Department of Psychiatry, University of South Dakota Sanford School of Medicine, South Dakota, USA, [39]Faculty of Medicine and Nursing, Department of Genetics, Physical Anthropology and Animal Physiology, University of the Basque Country (UPV/EHU), Leioa, Bizkaia, Spain, [40]Biocruces Bizkaia Health Research Institute, Barakaldo, Bizkaia, Spain, [41]CIBER de Diabetes y Enfermedades Metabólicas Asociadas (CIBEDEM), Madrid, Spain, [42]Department of Epidemiology and Biostatistics, School of Public Health, Imperial College London, London, UK, [43]MRC-PHE Centre for Environment and Health, School of Public Health, Imperial College London, London, UK., [44]Frazer Institute, The University of Queensland, Brisbane, Australia, [45]MRC Integrative Epidemiology Unit, University of Bristol, Bristol, UK, [46]Netherlands Twin Register, Department of Biological Psychology, Vrije Universiteit, Amsterdam, the Netherlands, [47]Amsterdam Reproduction & Development (AR&D) research institute, the Netherlands, [48]Population Health Sciences, Bristol Medical School, University of Bristol, Bristol, UK, [49]Center for Applied Genomics, Children's Hospital of Philadelphia, Philadelphia, USA, [50]Quantinuum Research LLC, San Diego, USA, [51]Division of Human Genetics, The Children's Hospital of Philadelphia, Philadelphia, USA, [52]ISGlobal, Institute for Global Health, Barcelona, Spain, [53]CIBER de Epidemiología y Salud Pública (CIBERESP), Madrid, Spain, [54]Universitat Pompeu Fabra (UPF), Barcelona, Spain, [55]Division of Endocrinology, Department of Pediatrics, Boston Children's Hospital, [56]Program in Medical and Population Genetics, Broad Institute of Harvard and MIT, Cambridge, MA, USA, [57]Department of Pediatrics, Harvard Medical School, Boston, MA, USA, [58]Institute for Molecular Bioscience, The University of Queensland, Brisbane, Australia, [59]MRC Lifecourse Epidemiology Unit, Faculty of Medicine, University of Southampton, Southampton, UK, [60]Department of Psychiatry, University of Vermont, Burlington, Vermont, [61]Division of Human Genetics, Children's Hospital of Philadelphia, Philadelphia, PA, USA., [62]Department of Genetics, University of Pennsylvania, Philadelphia, PA, USA, [63]Center for Spatial and Functional Genomics, Children's Hospital of Philadelphia, Philadelphia, PA, USA, [64]School of Biosciences and Medicine, University of Surrey, UK, [65]Division of Immunology, Immunity to Infection and Respiratory Medicine, School of Biological Sciences, The University of Manchester, Manchester Academic Health Science Centre, and Manchester University NHS Foundation Trust, Manchester, UK, [66]National Heart and Lung Institute, Imperial College London, UK, [67]MRC Epidemiology Unit, Institute of Metabolic Science, University of Cambridge School of Clinical Medicine, Cambridge, UK, [68]Department of Nutrition and Dietetics, Harokopio University of Athens, Athens, Greece., [69]Faculty of Medicine, Department of Medical Genetics, The University of British Columbia, Vancouver, Canada, [70]Center for Life Course Health Research, Faculty of Medicine, University of Oulu, Oulu, Finland, [71]Institute of Public Health and Clinical Nutrition, School of Medicine, University of Eastern Finland, Kuopio Campus, Finland, [72]Department of General Practice and Primary Health Care, University of Helsinki, Folkhälsan Research Center, Helsinki, Finland, [73]National University Singapore, Yong Loo Lin School of Medicine, Human Potential Translational Research Programme and Department of Obstetrics and Gynecology, Singapore, Singapore, [74]Singapore Institute for Clinical Sciences (SICS), Agency for Science, Technology and Research (A*STAR), Singapore, [75]Department of Epidemiology Research, Statens Serum Institut, Copenhagen, Denmark, [76]Department of Epidemiology Research, Statens Serum Institute, Copenhagen, Denmark, [77]The Generation R Study Group, Erasmus MC, University Medical Center Rotterdam, Rotterdam, the Netherlands, [78]Department of Pediatrics, Erasmus MC, University Medical Center Rotterdam, Rotterdam, the Netherlands, [79]School of Medical Science, Menzies Health Institute Queensland, Griffith University Gold Coast Campus, Southport, QLD, Australia, [80]Department of Obstetrics and Gynecology, Sahlgrenska University Hospital, Gothenburg, Sweden, [81]Department of Epidemiology, Erasmus MC, University Medical Center Rotterdam, Rotterdam, the Netherlands, [82]Center for Childhood Health, Islands Brygge 39-43, DK-2300, Copenhagen, Denmark, [83]Department of Pediatrics, Children's Hospital of Philadelphia, Philadelphia, PA, USA., [84]Department of Pediatrics, University of Pennsylvania Perelman School of Medicine, Philadelphia, USA, [85]Department of Genetics, University of Pennsylvania Perelman School of Medicine, Philadelphia, USA, [86]Division of Endocrinology and Diabetes, The Children's Hospital of Philadelphia, Philadelphia, USA, [87]Novo Nordisk Foundation Center for Basic Metabolic

Research, Faculty of Health and Medical Sciences, University of Copenhagen, Denmark, [88]Institute for Molecular Medicine, Finland (FIMM), University of Helsinki, Helsinki, Finland, [89]ISGlobal, Barcelona, Spain, [90]Universitat Pompeu Fabra, Barcelona, Spain, [91]Department of Child and Adolescent Psychiatry/Psychology, Erasmus MC, University Medical Centre, Rotterdam, The Netherlands, [92]Department of Pediatrics, University of California San Francisco School of Medicine, San Francisco, USA, [93]Children's Hospital of Philadelphia, Leonard Madlyn Abramson Research Center, Philadelphia, PA, USA, [94]Division of Endocrinology, Metabolism, and Molecular Medicine, Department of Medicine, Northwestern University Feinberg School of Medicine, Chicago, IL USA, [95]Center for Genetic Medicine, Northwestern University Feinberg School of Medicine, Chicago, IL USA, [96]Department of Anthropology, Northwestern University, Evanston, IL USA, [97]Department of Child and Adolescent Psychiatry, Psychosomatics and Psychotherapy, University Hospital Essen, University of Duisburg-Essen, Essen, Germany, [98]Center for Translational Neuro- and Behavioural Sciences, University Hospital Essen, Essen, Germany, [99]Institute and Clinic for Occupational, Social and Environmental Medicine, University Hospital, LMU Munich, Munich, Germany, [100]Research Unit of Population Health, University of Oulu, Oulu, Finland, [101]Department of Genetics and Bioinformatics, Domain of Health Data and Digitalisation, Norwegian Institute of Public Health, Oslo, Norway, [102]KG Jebsen Center for Diabetes Research, Department of Clinical Science, University of Bergen, Bergen, Norway, [103]Department of Pediatrics, Haukeland University Hospital, Bergen, Norway, [104]Department of Clinical Medicine - Department of Paediatrics, Aarhus University Hospital, Aarhus N, Denmark, [105]Diabetes Center, Massachusetts General Hospital, Boston, USA, [106]Department of Medicine, Universite de Sherbrooke, Sherbooke, Canada, [107]Institute of Nutritional Science, University of Potsdam, Nuthetal, Germany, [108]The First Affiliated Hospital of Jinan University, Guangzhou, China, [109]Department of Medicine Nephrology, Medical Faculty, Mannheim Heidelberg University, 68167 Mannheim, Germany, [110]Human Development & Health, Faculty of Medicine, University of Southampton, Southampton, UK, [111]The Children's Obesity Clinic, accredited European Centre for Obesity Management, Department of Pediatrics, Holbæk Hospital, Denmark, [112]RIKEN, Centre for Integrative Medical Sciences, Laboratory for Genomics of Diabetes and Metabolism, Yokohama, Japan, [113]Netherlands Twin Register, Department of Biological Psychology, Vrije Universiteit Amsterdam, Amsterdam, the Netherlands, [114]Netherlands Twin Register, Department of Biological Psychology, VU University, Amsterdam, the Netherlands, [115]Amsterdam Public Health, Amsterdam, the Netherlands, [116]Australian Centre for Precision Health, Unit of Clinical and Health Sciences, University of South Australia, Adelaide, Australia, [117]South Australian Health and Medical Research Institute, Adelaide, Australia, [118]Department of Obstetrics and Gynaecology, Sahlgrenska Academy, Institute of Clinical Science, University of Gothenburg, Gothenburg, Sweden, [119]Department of Genetics and Bioinformatics, Health Data and Digitalization, Norwegian Institute of Public Health, Oslo, Norway, [120]Institute of Health Sciences, University of Oulu, Oulu, Finland, [121]Biocenter Oulu, University of Oulu, Oulu, Finland, [122]Department of Epidemiology and Biostatistics, MRC Health Protection Agency (HPE) Centre for Environment and Health, School of Public Health, Imperial College London, UK, [123]Department of Children and Young People and Families, National Institute for Health and Welfare, Oulu, Finland, [124]Unit of Primary Care, Oulu University Hospital, Oulu, Finland, [125]Center for Medical Genetics and molecular Medicine, Haukeland University Hospital, Bergen, Norway, [126]Department of Clinical and Experimental Medicine, University of Surrey, Guildford, UK, [127]People-Centred Artificial Intelligence Institute, University of Surrey, Guildford, UK, [128]Department of Medicine, Imperial College London, London, UK, [129]Division of Gastroenterology, Hepatology and Nutrition, Cincinnati Children's Hospital Medical Center, Cincinnati, USA, [130]Lee Kong Chian School of Medicine, Nanyang Technological University, Singapore, Republic of Singapore, [131]University of Leipzig, Medical Faculty, Dept. of Women and Child Health, Pediatric Research Center, Leipzig, Germany, [132]Helmholtz Institute for Metabolic, Obesity and Vascular Research (HI-MAG) of the Helmholtz Zentrum München at the University of Leipzig and University Hospital Leipzig, Leipzig, Germany, [133]LIFE Child, University of Leipzig, Medical Faculty, LIFE–Leipzig Research Center for Civilization Diseases, Leipzig, Germany,, [134]Department of Child Health, School of Medicine, Cardiff Univeristy, Cardiff, UK, [135]Institute of Primary Care and Public Health, University of Lausanne, Lausanne, Switzerland, [136]Department of Computational Biology, University of Lausanne, Lausanne, Switzerland, [137]Swiss Institute of Bioinformatics, Lausanne, Switzerland, [138]Institute of Biomedicine, School of Medicine, University of Eastern Finland, Kuopio Campus, Finland; Department of Clinical

Physiology and Nuclear Medicine, Kuopio University Hospital, Kuopio, Finland; Foundation for Research in Health Exercise and Nutrition, Kuopio Research Institute of Exercise Medicine, Kuopio, Finland, [139]Department of Medicine, McMaster University, Hamilton, Ontario, Canada, [140]Division of Endocrinology, Department of Medicine, Creighton University, Omaha, USA, [141]Department of Clinical Chemistry, Fimlab Laboratories, Tampere 33520, Finland, [142]Department of Clinical Chemistry, Finnish Cardiovascular Research Center - Tampere, Faculty of Medicine and Health Technology, Tampere University, Tampere 33014, Finland, [143]Department of Cardiology, Heart Center, Tampere University Hospital, Tampere 33521, Finland, [144]Department of Medical Statistics, London School of Hygiene and Tropical Medicine, London, UK, [145]Wellcome Trust Centre for Human Genetics, University of Oxford, Oxford, UK, [146]Li Ka Shing Centre for Health Information and Discovery, The Big Data Institute, University of Oxford, Oxford, UK, [147]Department of Clinical Medicine, Faculty of Health and Medical Sciences,University of Copenhagen, Copenhagen, Denmark, [148]Nuffield Department of Population Health, Univeristy of Oxford, Oxford, UK, [149]Department of Medicine and Therapeutics, The Chinese University of Hong Kong, [150]Li Ka Shing Institute of Health Sciences, The Chinese University of Hong Kong, Hong Kong, China, [151]Hong Kong Institute of Diabetes and Obesity, The Chinese University of Hong Kong, Hong Kong, China, [152]Estonian Genome Center, University of Tartu, Tartu, Estonia, [153]Norwegian Institute of Public Health, Oslo, Norway, [154]Wellcome Centre for Human Genetics, University of Oxford, Oxford, UK, [155]Genentech, 1 DNA Way, South San Francisco, CA, USA, [156]Centre for Genetic Origins of Health and Disease (GOHaD), The University of Western Australia, Crawley, Australia, [157]Department of Clinical Medicine, Universityof Copenhagen, Copenhagen, Denmark, [158]Department of Medicine, Stanford UniversitySchool of Medicine, Stanford, California, USA, [159]Centre for Fertility and Health, NorwegianInstitute of Public Health, Oslo, Norway, [160]KG Jebsen Center for Genetic Epidemiology, Department of Public Health and Nursing, NTNU, Norwegian University of Science and Technology, Norway, [161]Institute of Clinical Medicine, Faculty of Medicine, University of Oslo, Norway., [162]Department of Genetics, University of North Carolina, Chapel Hill, NC, USA, [163]Department of Clinical Epidemiology, Leiden University Medical Center, 2333 ZA Leiden, The Netherlands, [164]Department of Public Health and Primary Care, Leiden University Medical Center, 2333 ZA Leiden, The Netherlands, [165]Centre for Genetics and Genomics Verusus Arthritis, Centre for Musculoskeletal Research, The University of Manchester, Manchester, UK, [166]Department of Public and Occupational Health, Amsterdam University Medial Center, University of Amsterdam, Amsterdam, [167]Dept of Pediatrics, University of Iowa, Iowa City, IA 52240 USA, [168]Department of Genes and Environment, Division of Epidemiology, Norwegian Institute of Public Health, Oslo, Norway, [169]Centre for Population Health Research, University of Turku and Turku University Hospital, Turku, Finland, [170]Research Centre of Applied and Preventive Cardiovascular Medicine, University of Turku, Turku, Finland, [171]Department of Pediatrics and Adolescent Medicine, University of Turku and University Hospital of Turku, Turku, Finland, [172]Mohn Center for Diabetes Precision Medicine, Department of Clinical Science, University of Bergen, NO-5020 Bergen, Norway, [173]Children and Youth Clinic, Haukeland University Hospital, NO-5021 Bergen, Norway, [174]Department of Clinical Research, Research Unit for Obstetrics and Gynecology, University of Southern Denmark, Odense, Denmark, [175]William Harvey Research Institute, Barts and the London School of Medicine and Dentistry, Queen Mary University of London, London, UK, [176]Division of Pediatric Endocrinology, Diabetes, and Metabolism, Department of Pediatrics, Columbia University Medical Center, New York, USA, [177]Department of Paediatrics, University of Cambridge School of Clinical Medicine, Cambridge, UK, [178]Paavo Nurmi Centre and Unit for Health and Physical Activity, University of Turku, Turku, Finland, [179]School of Medicine and Public Health, University of Newcastle, Newcastle, NSW, Australia, [180]Hunter Medical Research Institute, Newcastle, NSW, Australia, [181]Department of Psychiatry and Behavioral Sciences, College of Medicine, State University of New York Downstate Health Sciences University, Brooklyn, New York, USA, [182]Population, Policy, Practice. Great Ormond Street Institute of Child Health, University College London, London, UK, [183]Institute of Molecular Medicine, Helsinki, Finland, [184]Institut Pasteur de Lille, CNRS, University of Lille, Lille, France., [185]Institute of Biochemistry and Genetics, Ufa Federal Research Centre, Russian Academy of Sciences, Ufa, Russian Federation, [186]Department of Clinical Physiology and Nuclear Medicine, Turku University Hospital, Turku, Finland, [187]Centre for Cardiovascular Science, Queen's Medical Research Institute, University of Edinburgh, Edinburgh, UK, [188]Medical Research Council Integrative Epidemiology Unit at the University of Bristol, Bristol,

UK, [189]Department of Epidemiology and Biostatistics, MRC‚ÄìPHE Centre for Environment & Health, School of Public Health, Imperial College London, London, UK, [190]Department of Psychology, Mid Sweden University, Östersund, Sweden, [191]Herbert Wertheim School of Public Health, University of California San Diego, La Jolla, CA USA, [192]Department of Genetics, Harvard Medical School, Boston, USA, [193]Center for Basic and Translational Obesity Research, Boston Children's Hospital, Boston, USA, [194]Singapore Eye Research Institute, Singapore, [195]Yong Loo Lin School of Medicine, National University of Singapore, Singapore, [196]Liggins Institute, University of Auckland, Auckland, NZ, [197]Research Unit of Population Health, Faculty of Medicine, University of Oulu, Oulu, Finland, [198]Institute of Epidemiology, Helmholtz Zentrum München - German Research Center for Environmental Health, Neuherberg, Germany, [199]Department of Obstetrics and Gynecology, Erasmus MC, University Medical Center, Rotterdam, the Netherlands, [200]Max Planck Institute for Psycholinguistics, Nijmegen, the Netherlands, [201]Population Health Research Institute, St George's, University of London, London, UK, [202]IMIM (Hospital del Mar Medical Research Institute), Barcelona, Spain, [203]Saw Swee Hock School of Public Health, National University of Singapore and National University Health System, Singapore, Singapore, [204]National University of Singapore, Singapore, Singapore, [205]Division of Metabolic and Nutritional Medicine, Dr. von Hauner Children's Hospital, University of Munich Medical Center, Munich, Germany, [206]European Centre for Environment and Human Health, University of Exeter, Truro, UK, [207]Department of Internal Medicine, Erasmus Medical Centre, Rotterdam, The Netherlands, [208]Department of Epidemiology, Erasmus MC University Medical Center, Rotterdam, The Netherlands, [209]Department of Public Health, Oxford University, Oxford, UK, [210]Center for Diabetes Research, Department of Clinical Science, University of Bergen, Bergen, Norway, [211]Institute of Medical Informatics, Biometry and Epidemiology, Chair of Epidemiology, Ludwig Maximilians University, Munich, Germany, [212]Helmholtz Center Munich, Institute of Epidemiology, Neuherberg, Germany, [213]Institute of Medical Statistics and Epidemiology, Technical University Munich, Munich, Germany, [214]Institute for Molecular Medicine Finland (FIMM), HiLIFE, University of Helsinki, Helsinki, Finland, [215]Netherlands Twin Register, Department of Biological Psychology, Vrije Universiteit, Amsterdam, Amsterdam, Netherlands, [216]Centre for Global Health Research, Usher Institute, University of Edinburgh, Teviot Place, Edinburgh, EH8 9AG, Scotland, [217]Institute of Translational Genomics, Helmholtz Zentrum München – German Research Center for Environmental Health, 85764 Neuherberg, Germany, [218]Technical University of Munich (TUM) and Klinikum Rechts der Isar, TUM School of Medicine, Ismaninger Str. 22, 81675 Munich, Germany, [219]Division of Gastroenterology, Hepatology and Nutrition, Children's Hospital of Philadelphia, PA, USA, [220]Center for Prevention of Preterm Birth, Perinatal Institute, Cincinnati Children's Hospital Medical Center, Cincinnati, Ohio, USA, [221]March of Dimes Prematurity Research Center Ohio Collaborative, Cincinnati, USA, [222]Division of Human Genetics, Cincinnati Children's Hospital Medical Center, Cincinnati, Ohio, USA, [223]Department of Pediatrics, University of Cincinnati College of Medicine, Cincinnati, Ohio, USA

Supplementary Note 2: Cohort description, acknowledgements and funding

Avon Longitudinal Study of Parents and Children (ALSPAC)

Pregnant women resident in Avon, UK with expected dates of delivery between 1st April 1991 and 31st December 1992 were invited to take part in the study[1,2]. The initial number of pregnancies enrolled was 14,541. Of the initial pregnancies, there was a total of 14,676 foetuses, resulting in 14,062 live births and 13,988 children who were alive at 1 year of age. When the oldest children were approximately 7 years of age, an attempt was made to bolster the initial sample with eligible cases who had failed to join the study originally. As a result, when considering variables collected from the age of seven onwards (and potentially abstracted from obstetric notes) there are data available for more than the 14,541 pregnancies mentioned above: The number of new pregnancies not in the initial sample (known as Phase I enrolment) that are currently represented in the released data and reflecting enrolment status at the age of 24 is 906, resulting in an additional 913 children being enrolled (456, 262 and 195 recruited during Phases II, III and IV respectively). The total sample size for analyses using any data collected after the age of seven is therefore 15,447 pregnancies, resulting in 15,658 foetuses. Of these 14,901 children were alive at 1 year of age.
Full instructions for applying for data access can be found here:
http://www.bristol.ac.uk/alspac/researchers/access/.

Please note that the study website contains details of all the data that is available through a fully searchable data dictionary and variable search tool:
http://www.bristol.ac.uk/alspac/researchers/our-data/

From birth to approximately five years of age, length and weight measurements were abstracted from health visitor records. Health visits form part of the standard childcare in the UK and data was abstracted from records at approximately six weeks, 10, 21, and 48 months of age. From ages 4 months to 5 years a sub-study (called "Child in Focus") included a random 10% of the cohort and recorded data across eight research clinics. Length/height and weight were also measured at these clinics. From age 7 onwards, all children were invited to annual research clinics in which height and weight were measured. Furthermore, parent-reported height and weight was also recorded via questionnaires across the time course. BMI was derived from the length/height and weight measures (mean of 9 measures per participant) as weight (kg) divided by the square of height (m). Given the inclusion of measured and self-reported height and weight data, we additionally included an indicator of the source of data as a covariable in the LMMs (clinic/health visit vs. questionnaire). All participants with at least one measure of BMI was included in the study, however, we excluded those who were part of a multiple birth (i.e. twins, triplets etc. n=524).

Ethical approval for the study was obtained from the ALSPAC Ethics and Law Committee and the Local Research Ethics Committees. Informed consent for the use of data collected via questionnaires and clinics was obtained from participants following the recommendations of the ALSPAC Ethics and Law Committee at the time.

We are extremely grateful to all the families who took part in this study, the midwives for their help in recruiting them, and the whole ALSPAC team, which includes interviewers, computer and laboratory technicians, clerical workers, research scientists, volunteers, managers, receptionists and nurses.

The UK Medical Research Council and Wellcome (Grant ref: 217065/Z/19/Z) and the University of Bristol provide core support for ALSPAC. A comprehensive list of grants funding is available on the ALSPAC website (http://www.bristol.ac.uk/alspac/external/documents/grant-acknowledgements.pdf). Genome-wide genotyping data was generated by Sample Logistics and Genotyping Facilities at Wellcome Sanger Institute and LabCorp (Laboratory Corporation of America) using support from 23andMe. This publication is the work of the authors and Kimberley Burrows will serve as guarantors for the contents of this paper.

For the purpose of Open Access, the author has applied a CC BY public copyright license to any Author Accepted Manuscript version arising from this submission.

Northern Finland Birth Cohorts born in 1966 (NFBC1966)

The Northern Finland Birth Cohort study 1966 (NFBC1966) includes 12,058 live born individuals, of European descent, with expected dates of birth during 1966 in the two northernmost provinces of Finland, Oulu and Lapland[3]. The data on all cohort members were prospectively collected since pregnancy and supplemented at the ages of 1, 14, 31 and 46 years[4]. Height and weight growth measurements were obtained from communal child health clinics[5]. All those living in northern Finland or in the capital area were invited to a clinical examination and blood sampling at age 31 years. DNA was extracted from 5753 individuals and Illumina's HumanCNV370-Duo DNA Analysis BeadChip was used to obtain genome-wide genetic data[6].

Prior to growth trajectory modelling, we excluded participants born pre-term (n=765) and non-singletons (n=197). We also identified the sibling pairs of this cohort that were born at different times, and one participant per sibling pair was excluded (n=17).

Approval for the studies was granted by the Northern Ostrobothnia Hospital District Ethical

Committee 94/2011 (12.12.2011), Finland in accordance with the declaration of Helsinki. Mothers gave their informed consent in the beginning of the NFBC1966 data collection. Written informed consent has been obtained from the cohort participants in the 31- and 46-year data collections.

We thank all cohort members and researchers who participated in the study. We also wish to acknowledge the work of the NFBC project center. NFBC1966 31-year follow-up received financial support from University of Oulu Grant no. 65354, Oulu University Hospital Grant no. 2/97, 8/97, Ministry of Health and Social Affairs Grant no. 23/251/97, 160/97, 190/97, National Institute for Health and Welfare, Helsinki Grant no. 54121, Regional Institute of Occupational Health, Oulu, Finland Grant no. 50621, 54231. NFBC1966 46yr follow-up received financial support from University of Oulu Grant no. 24000692, Oulu University Hospital Grant no. 24301140, ERDF European Regional Development Fund Grant no. 539/2010 A31592. This work was partially supported by the MRC Centre for Environment and Health, which is currently funded by the Medical Research Council (MR/S019669/1, 2019-2024).

NFBC1966 data are available from the University of Oulu, Infrastructure for Population Studies. Permission to use the data can be applied for research purposes via an electronic material request portal. In the use of data, we follow the EU general data protection regulation (679/2016) and the Finnish Data Protection Act. The use of personal data is based on a cohort participant's written informed consent in their latest follow-up study, which may cause limitations to its use. Please contact the NFBC project center (NFBCprojectcenter(at)oulu.fi) and visit the cohort website (www.oulu.fi/nfbc) for more information.

Northern Finland Birth Cohorts born in 1986 (NFBC1986)

The Northern Finland Birth Cohort 1986 (NFBC1986) includes 9,432 live born children with expected dates of birth between 1st July 1985 and 30th June 1986 in the two northernmost provinces of Finland, Oulu and Lapland[7,8]. The cohort has been followed up since early pregnancy until adulthood[9]. Growth measurements were obtained from communal child health clinics. All those alive with known address were invited to a clinical examination at the age of 15 to 16 years. At this age, blood samples were drawn and DNA was extracted for 6,266 subjects.

Prior to growth trajectory modelling, we excluded pre-term babies (n=496) as well as twins and triplets (n=132). From the remaining sample, we further identified one sibling pair that was born at different times, and the child with less height and weight measurements was excluded. Finally, one child with extremely fast BMI growth from early on was excluded from the sample as the trajectory was not comparable to other children.

Approval for the studies was granted by the Northern Ostrobothnia Hospital District Ethical Committee 108/2017 (15.1.2018), Finland in accordance with the declaration of Helsinki. Participants provided written informed consent in data collections carried out when they were aged 15 to 16 and 33 to 35 years.

We thank all cohort members and researchers who have participated in the study. We also wish to acknowledge the work of the NFBC project center. The cohort received financial support from the following grants EU QLG1-CT-2000-01643 (EUROBLCS) Grant no. E51560, NorFA Grant no. 731, 20056, 30167, USA / NIH 2000 G DF682 Grant no. 50945, and from Academy of Finland EGEA-project (285547).

NFBC1986 data are available from the University of Oulu, Infrastructure for Population Studies. Permission to use the data can be applied for research purposes via an electronic material request portal. In the use of data, we follow the EU general data protection regulation (679/2016) and the Finnish Data Protection Act. The use of personal data is based on a cohort participant's written informed consent in their latest follow-up study, which may cause limitations to its use. Please contact the NFBC project center (NFBCprojectcenter(at)oulu.fi) and visit the cohort website (www.oulu.fi/nfbc) for more information.

CHOP

The CHOP cohort is a random sampling of the population of children who come for care at the Children's Hospital of Philadelphia starting in 2006 and continuing presently[10]. If patients choose to enroll in the study, their fully de identified and encrypted electronic medical records are available for study. Heights and weights used to calculate BMI in this study were obtained from electronic medical records. Self-reported ethnicity was used to define the African American and European American subsets used in all analyses. Self-reported ethnicity was confirmed by principal components analysis. The CHOP study was genotyped at the Center for Applied Genomics at the Children's Hospital of Philadelphia. The Research Ethics Board of CHOP approved the study, and written informed consent was obtained from all subjects.

CHOP-related data are available upon request from Hakon Hakonarson (hakonarson@chop.edu; response timeframe: one month). Please note that one limitation of the request process is the transfer of data under a material transfer agreement.

The authors thank the network of primary care clinicians and the patients and families for their contribution to this project and to clinical research facilitated by the Pediatric Research Consortium [PeRC]-The Children's Hospital of Philadelphia. R. Chiavacci, E. Dabaghyan, A. [Hope] Thomas, K. Harden, A. Hill, C. Johnson-Honesty, C. Drummond, S. Harrison, F. Salley, C. Gibbons, K. Lilliston, C. Kim, E. Frackelton, F. Mentch, G. Otieno, K. Thomas, C. Hou, K. Thomas and M.L. Garris provided expert assistance with genotyping and/or data collection and management. The authors would also like to thank S. Kristinsson, L.A. Hermannsson and A. Krisbjörnsson of Raförninn ehf for extensive software design and contributions. This research was financially supported by an Institute Development Award from the Children's Hospital of Philadelphia, a Research Development Award from the Cotswold Foundation, the Daniel B. Burke Endowed Chair for Diabetes Research, the Children's Hospital of Philadelphia Endowed Chair in Genomic Research and NIH grant R01 HD056465.

OBE

The OBésité de l'Enfant (OBE) cohort includes children with early-onset, familial obesity, who were born in France. Most of them were recruited by the CNRS 8199 unit in 1998. A small part of these children were patients of Toulouse Children's Hospital. Height and weight measurements were taken by the participant's GP or pediatrician. The study protocols were approved by local ethics committees. Oral assent from children was obtained and parents (or legal guardians) signed an informed consent form.

OBE-related data are available upon request from Philippe Froguel (p.froguel@imperial.ac.uk; response timeframe: one month). Please note that one limitation of the request process is the transfer of data under a material transfer agreement.

The authors would like to thank the French National Research Agency (Agence Nationale de la Recherche [ANR]-10-LABX-46 [European Genomics Institute for Diabetes] and ANR-10-EQPX-07-01 [Lille Integrated Genomics Advanced Network for personalized medicine] to A.B. and P.F.), European Research Council (OpiO 101043671 to A.B.), the European Union's Horizon Europe Research and Innovation Programme (OBELISK grant agreement 101080465 to A.B. and P.F.), and the National Center for Precision Diabetic Medicine (PreciDIAB to A.B. and P.F), which is jointly supported by the French National Agency for Research (ANR-18-IBHU-0001), European Regional Development Fund, Hauts-de-France Regional Council, and the European Metropolis of Lille. The authors also thank the France Génomique consortium (ANR-10-INBS-009).

Supplementary Note 3: Example process from the EGGLA BMI modelling framework

While the framework described below is specific to the current study exploring the GWAS of longitudinal BMI trajectories and estimated phenotypes, the framework can serve as an exemplar for other consortium efforts to analyse other non-linear longitudinal traits.

To undertake LMM analysis of BMI, analysts are required to load in longitudinal data in long format with the following mandatory columns: individual ID, sex, height measurement (cm), weight measurement (kg), and age at measurement (days or years). Additional cohort specific covariates may also be included.

A comprehensive protocol has been written to allow the user to set up the environment and work through the analysis pipeline. This is available at: https://m.canouil.dev/eggla/articles/eggla.html. The online protocol contains a series of vignettes containing the code used to perform the model diagnostics framework (https://m.canouil.dev/eggla/articles/models-diagnostics.html), a plot to aid in model selection (https://m.canouil.dev/eggla/articles/model-selection.html), analyses and plots specific to the chosen model from the diagnostics framework (in this case specific to the cubic spline model) (https://m.canouil.dev/eggla/articles/run-cubic-splines.html), and finally characterisation of the AP and AR (https://m.canouil.dev/eggla/articles/adiposity-peak-rebound.html). See each vignette for examples of output using simulated data.

The chosen best model selected from the output of the vignettes above was taken forward for model refinement (see methods of main paper). Finally, two EGGLA R package[13] functions; run_eggla_lmm() and run_eggla_gwas() were used to perform the LMM and GWAS using the final chosen model (cubic spline function in the fixed effects with cubic slope function random effects).

The EGGLA[13] function run_eggla_lmm() is a wrapper used to perform the following analysis stages: 1) cleaning and formatting of BMI data using the growthcleanr R package for longitudinal data cleaning, 2) deriving BMI from cleaned height and weight data and formatting of the dataframe for analysis, 3) run the LMM for males and females separately, 4) plotting of model residuals, 5) deriving slopes and AUCs for defined linear time intervals of the curve, 6) predict BMI and derive age and BMI at AP and AR, 7) detection of outlier individuals for each of the estimated phenotypes and output of correlations between latent phenotypes, 8) formatting the derived phenotypes into a dataset ready for GWAS. The run_eggla_lmm() function allows for further refinement of models where applicable including: addition of covariates, choice of the number and placement of knot points, choice of the linear time periods for derivation of slopes and AUCs, inclusion of correlation structure and complexity of random effects. After specifying the parameters of the chosen model (knot points, time intervals, covariates, number of iterations, and parameters for outliers) and running the model using the run_eggla_lmm() R function, several outputs are provided to aid the analyst in making a final checks; these outputs include plots of residuals, the model coefficients, the model call, a table of pairwise correlations between all derived phenotypes and a list of outliers identified for each of the latent variables. This allows the analyst to make decisions about model fit, outlier inclusion or exclusion and inform downstream analysis based on the extent of the correlation between the derived phenotypes. Finally, individual level derived phenotypes for each specified time-period are output as comma-separated files. For instance a .csv file will contain all individuals (males and females have a separate .csv file) and their slopes for infancy, childhood, late childhood, and adolescence. The csv files can then be called by the run_eggla_gwas() R function or the sex stratified files can be combined and included in the analysts preferred software for GWAS.

The run_eggla_gwas() function performs GWAS analysis that can be implemented by any cohort with unrelated individuals (any complex data structures, such as related family structure, will need to use alternative GWAS software), where genetic dosage data is available in VCF file format. The function analyses VCF files through software packages BFCtools (v.≥1.16) (https://github.com/samtools/BCFtools) and PLINK2 (v.≥2.0) (https://www.cog-genomics.org/plink/2.0) and therefore requires access to these packages. The following actions are performed using the run_eggla_gwas() function: 1) format input data and perform checks for ID matching, and removing outliers on a complete case basis if required, 2) format VCFs through BCFtools and annotate if requested, 3) perform GWAS using PLINK2, 4) extract information from VCF INFO field if requested, and 5) allows for further options including: annotation files to add rsID and gene symbols.

Supplementary Note 4: Linear mixed modelling

A simple approach is to model a repeated continuous outcome (e.g. BMI) as a function of time within a linear mixed model (LMM). LMM has an advantage over other methods, such as repeated measures ANOVA, by allowing missing data within individuals, and unbalanced study designs where either the number of measures or the timing of the measurements differs between individuals, as well as fitting and testing covariance structures[11].

A basic random-slope LMM with a single continuous outcome such as BMI as a linear function of time can be written as:

$$Y_{it} = \beta_0 + \beta_1 X_{it} + \upsilon_{0i} + \upsilon_{1i} X_{it} + \varepsilon_{it} \qquad (1)$$

Where Y_{it} is a single outcome measured for individual *i* at time *t* and is assumed to be independent between individuals. The fixed coefficients, β_0 and β_1 represent the average intercept and slope respectively. The random coefficients represent the deviation from the average intercept (υ_{0i}) and slope (υ_{1i}) for individual *i*. The random effects are assumed to follow a bivariate normal distribution with mean zero and covariance matrix Ω_υ. Residual errors ε_{it} are assumed to be independently identically normally (i.i.d) distributed with variance σ_{e0}^2.

The LMM model above assumes a linear change in the continuous outcome with increasing time. However, growth is nonlinear for many biological characteristics, such as BMI across early life. BMI throughout infancy, childhood, and adolescence follows a complex pattern of change including the appearance of the adiposity peak during infancy and then a rebound during childhood (see Figure 1 in the main text). This nonlinear change can be incorporated into LMM models through the addition polynomial functions of time in the fixed and random components of the model. A quadratic function for instance can induce a curve with a single turn, while a cubic function can involve up to two turns. More simple polynomials may not adequately describe the turning points in the underlying data, while more complex polynomials can produce artefactual turns in the curve and can particularly fit poorly at extremes where data is more sparse. A more flexible approach to model complex curves such as BMI is through using spline functions, joined at knot points dispersed across time. Smooth functional polynomials (usually of low order) are chosen to fit the measures between consecutive knots[12].

Supplementary References

1. Boyd, A. *et al.* Cohort Profile: The 'Children of the 90s'—the index offspring of the Avon Longitudinal Study of Parents and Children. *Int. J. Epidemiol.* **42**, 111–127 (2013).
2. Fraser, A. *et al.* Cohort Profile: the Avon Longitudinal Study of Parents and Children: ALSPAC mothers cohort. *Int J Epidemiol* **42**, 97–110 (2013).
3. University of Oulu., 1966. Northern Finland Birth Cohort 1966. https://etsin.fairdata.fi/dataset/716939c3-7a2a-4b6a-91f3-92aca09bc52d (1966).
4. Nordström, T. *et al.* Cohort Profile: 46 years of follow-up of the Northern Finland Birth Cohort 1966 (NFBC1966). *International Journal of Epidemiology* **50**, 1786–1787j (2021).
5. Sovio, U. *et al.* Genetic Determinants of Height Growth Assessed Longitudinally from Infancy to Adulthood in the Northern Finland Birth Cohort 1966. *PLoS Genet* **5**, e1000409 (2009).
6. Sabatti, C. *et al.* Genome-wide association analysis of metabolic traits in a birth cohort from a founder population. *Nat Genet* **41**, 35–46 (2009).
7. Järvelin, M. R., Hartikainen-Sorri, A. L. & Rantakallio, P. Labour induction policy in hospitals of different levels of specialisation. *Br J Obstet Gynaecol* **100**, 310–315 (1993).
8. Järvelin, M. R. *et al.* Ecological and individual predictors of birthweight in a northern Finland birth cohort 1986. *Paediatr Perinat Epidemiol* **11**, 298–312 (1997).
9. University of Oulu., 1986. Northern Finland Birth Cohort 1986. https://etsin.fairdata.fi/dataset/f22c6599-2293-42bd-b65a-1a77945ed613 (1986).
10. Connolly, J. J., Glessner, J. T., Li, D., Sleiman, P. M. & Hakonarson, H. The Center for Applied Genomics at The Children's Hospital of Philadelphia – Pediatric Perspectives on Genomic Medicine. *The Journal of Precision Medicine* https://www.thejournalofprecisionmedicine.com/the-journal-of-precision-medicine/the-center-for-applied-genomics-at-the-childrens-hospital-of-philadelphia-pediatric-perspectives-on-genomic-medicine/ (2020).
11. Laird, N. M. & Ware, J. H. Random-effects models for longitudinal data. *Biometrics* **38**, 963–974 (1982).

12. Perperoglou, A., Sauerbrei, W., Abrahamowicz, M. & Schmid, M. A review of spline function procedures in R. *BMC Medical Research Methodology* **19**, 46 (2019).
13. Canouil, M., Warrington, N., Burrows, K. & Heiskala, A. eggla: Early Growth Genetics Longitudinal Analysis. Zenodo https://doi.org/10.5281/zenodo.10594717 (2024).
