# Supplementary material for: A framework for conducting GWAS using repeated measures data with an application to childhood BMI
Source: Nat Commun. 2024 Nov 20;15:10067. doi: 10.1038/s41467-024-53687-3 (PMC11579382; doi:10.1038/s41467-024-53687-3)
Supplement: Supplementary file 3 — Description of additional supplementary files [file 41467_2024_53687_MOESM3_ESM.pdf]

Description of Additional Supplementary Files

File Name: Supplementary Data 1.xlsx
Description: Descriptive statistics of each cohort included in the analysis

File Name: Supplementary Data 2.xlsx
Description: Summary of height, weight and body mass index data removed through the quality control implemented in growthcleanr

File Name: Supplementary Data 3.xlsx
Description: Model fit parameters from each cohort for each of the methods.

File Name: Supplementary Data 4.xlsx
Description: Correlation between each of the estimated phenotypes in each of the cohorts.

File Name: Supplementary Data 5.xlsx
Description: Correlation between each of the estimated phenotypes in ALSPAC and NFBC1986 in males and females where the within-individual variation (i.e. the random effects and correlation structure) is modelled differently.

File Name: Supplementary Data 6.xlsx
Description: Estimated genome-wide genetic correlations between the twelve estimated phenotypes summarising growth across early life and BMI in childhood and adulthood.

File Name: Supplementary Data 7.xlsx
Description: Summary statistics for the genome-wide significant SNPs ($P<5x10^{-8}$) from the inverse-variance-weighted fixed-effects meta-analysis of each estimated phenotype, including a two-sided association P-value.

File Name: Supplementary Data 8.xlsx
Description: Summary statistics for the genome-wide significant SNPs ($P<5x10^{-8}$) from the inverse-variance-weighted fixed-effects meta-analysis of each estimated phenotype and replication in the African American subset from CHOP, including a two-sided association P-value.

File Name: Supplementary Data 9.xlsx
Description: Summary statistics from the inverse-variance-weighted fixed-effects meta-analysis of each estimated phenotype for the previously identified childhood obesity and BMI associated SNPs, including a two-sided association P-value.
