# Supplementary material for: A framework for conducting GWAS using repeated measures data with an application to childhood BMI
Source: Nat Commun. 2024 Nov 20;15:10067. doi: 10.1038/s41467-024-53687-3 (PMC11579382; doi:10.1038/s41467-024-53687-3)
Supplement: Supplementary file 13 — Reporting Summary [file 41467_2024_53687_MOESM13_ESM.pdf]

nature portfolio

Corresponding author(s): Nicole M Warrington and Sylvain Sebert

Last updated by author(s): Sep 9, 2024

Reporting Summary

Nature Portfolio wishes to improve the reproducibility of the work that we publish. This form provides structure for consistency and transparency in reporting. For further information on Nature Portfolio policies, see our Editorial Policies and the Editorial Policy Checklist.

Statistics

For all statistical analyses, confirm that the following items are present in the figure legend, table legend, main text, or Methods section.

n/a	Confirmed	
☐	☒	The exact sample size (*n*) for each experimental group/condition, given as a discrete number and unit of measurement
☐	☒	A statement on whether measurements were taken from distinct samples or whether the same sample was measured repeatedly
☐	☒	The statistical test(s) used AND whether they are one- or two-sided<br>*Only common tests should be described solely by name; describe more complex techniques in the Methods section.*
☐	☒	A description of all covariates tested
☐	☒	A description of any assumptions or corrections, such as tests of normality and adjustment for multiple comparisons
☐	☒	A full description of the statistical parameters including central tendency (e.g. means) or other basic estimates (e.g. regression coefficient) AND variation (e.g. standard deviation) or associated estimates of uncertainty (e.g. confidence intervals)
☐	☒	For null hypothesis testing, the test statistic (e.g. *F*, *t*, *r*) with confidence intervals, effect sizes, degrees of freedom and *P* value noted<br>*Give P values as exact values whenever suitable.*
☒	☐	For Bayesian analysis, information on the choice of priors and Markov chain Monte Carlo settings
☐	☒	For hierarchical and complex designs, identification of the appropriate level for tests and full reporting of outcomes
☐	☒	Estimates of effect sizes (e.g. Cohen's *d*, Pearson's *r*), indicating how they were calculated

Our web collection on statistics for biologists contains articles on many of the points above.

Software and code

Policy information about availability of computer code

Data collection	Data collection was performed by the investigators of each contributing EGG Consortium study. We provide detailed supplementary tables with characteristics of each study and references to study descriptions in the Methods section of the manuscript.
Data analysis	The source code for the R package, EGGLA, developed and used for data analysis in the study is available at: https://m.canouil.dev/eggla/index.html. The analysis within each cohort was performed up to version v0.20.0.<br>We used PLINK (version 2.0), REGENIE (version 3.2.6), EasyQC2 (v. 1.1.1.b5), GWAMA (version v.2.1), LocusZoomm (version 0.14.0), UCSC Lift Genome Annotations browser tool, Linkage Disequilibrium (LD) score regression (version 1.0.1) and R (each cohort analyst used their own version) to analyse the data and produce plots for this manuscript. A full description of software used in this paper is provided in the Methods section, along with references to the relevant journal articles.

For manuscripts utilizing custom algorithms or software that are central to the research but not yet described in published literature, software must be made available to editors and reviewers. We strongly encourage code deposition in a community repository (e.g. GitHub). See the Nature Portfolio guidelines for submitting code & software for further information.

Data

Policy information about availability of data

All manuscripts must include a data availability statement. This statement should provide the following information, where applicable:

- Accession codes, unique identifiers, or web links for publicly available datasets
- A description of any restrictions on data availability
- For clinical datasets or third party data, please ensure that the statement adheres to our policy

To access phenotype and genotype data from individual cohorts participating in the EGG consortium, the cohorts should be contacted directly as each cohort has different data access policies. Further Full details of each participating cohort can be found in the Supplementary TextNote 2. Briefly,
- ALSPAC: Full instructions for applying for data access can be found here: http://www.bristol.ac.uk/alspac/researchers/access/.
- CHOP: CHOP-related data are available upon request from Hakon Hakonarson (hakonarson@chop.edu; response timeframe: one month). Please note that one limitation of the request process is the transfer of data under a material transfer agreement.
- NFBC1966: Please, contact the NFBC project center (NFBCprojectcenter(at)oulu.fi) and visit the cohort website (www.oulu.fi/nfbc).
- NFBC1986: Please, contact the NFBC project center (NFBCprojectcenter(at)oulu.fi) and visit the cohort website (www.oulu.fi/nfbc).
- OBE: OBE-related data are available upon request from Philippe Froguel (p.froguel@imperial.ac.uk; response timeframe: one month). Please note that one limitation of the request process is the transfer of data under a material transfer agreement.

GWAS summary statistics from this study are available via the EGG website (http://egg-consortium.org/longitudinal_growth.html). Source data are provided with this paper.

Research involving human participants, their data, or biological material

Policy information about studies with human participants or human data. See also policy information about sex, gender (identity/presentation), and sexual orientation and race, ethnicity and racism.

Reporting on sex and gender	Linear mixed modeling was stratified by sex within each cohort. The genome-wide association analysis included sex within each cohort as a covariate.
Reporting on race, ethnicity, or other socially relevant groupings	In this study we report in terms of ethnicity; for instance, in ALSPAC and CHOP European American and African American cohorts' ethnicity was self-reported. However, as part of the genome-wide data quality control processes applied to each participating cohort, self-reported ethnicity was confirmed by principal components analysis and principal components were subsequently controlled for as confounding variables in genome-wide association analysis.
Population characteristics	ALSPAC is a birth cohort where pregnant women resident in Avon, UK with expected dates of delivery between 1st April 1991 and 31st December 1992 were invited to take part in the study. NFBC1966 is a birth cohort with expected dates of birth during 1966 in the two northernmost provinces of Finland, Oulu and Lapland. Similarly, NFBC1986 is also a birth cohort of pregnant mothers in the same two provinces but with expected dates of birth between 1st July 1985 and 30th June 1986. The CHOP cohort is a random sample of the population of children who come for care at the Children's Hospital of Philadelphia, starting in 2006 and continuing presently. The OBésité de l'Enfant (OBE) cohort includes children with early-onset, familial obesity who were born in France, with most individuals recruited in 1998. Data from individuals aged between 2 weeks and 18 years was included in the analyses for all cohorts. 45-54% of the individuals were male in each of the cohorts. Participants were not selected on the basis of disease status. All cohort specific details are provided in the supplementary material.
Recruitment	See above for recruitment detail. Specific details for each cohort are given in the supplementary material and references.
Ethics oversight	ALSPAC: Ethical approval for the study was obtained from the ALSPAC Ethics and Law Committee and the Local Research Ethics Committees.<br><br>NFBC1966: Approval for the studies was granted by the Northern Ostrobothnia Hospital District Ethical Committee 94/2011 (12.12.2011), Finland in accordance with the declaration of Helsinki.<br><br>NFBC1986: Approval for the studies was granted by the Northern Ostrobothnia Hospital District Ethical Committee 108/2017 (15.1.2018), Finland in accordance with the declaration of Helsinki.<br><br>CHOP: The Research Ethics Board of CHOP approved the study.<br><br>OBE: The study protocols were approved by local ethics committees.

Note that full information on the approval of the study protocol must also be provided in the manuscript.

Field-specific reporting

Please select the one below that is the best fit for your research. If you are not sure, read the appropriate sections before making your selection.

☒ Life sciences ☐ Behavioural & social sciences ☐ Ecological, evolutionary & environmental sciences

For a reference copy of the document with all sections, see nature.com/documents/nr-reporting-summary-flat.pdf

Life sciences study design

All studies must disclose on these points even when the disclosure is negative.

Sample size	The analysis for the growth modeling comprised 34,818 females (ranging from 308 to 10,814 per cohort) and 36,518 males (ranging from 252 to 12,002 per cohort). The GWAS analysis comprised 12,685 females (ranging from 261 to 3,375 per cohort) and 12,955 males (ranging from 229 to 3,532 per cohort). We used all available data from each of the cohorts without conducting sample-size calculations because the purpose for this analysis was to examine the performance of the statistical methods across a range of cohorts.
Data exclusions	We excluded individuals who were part of a multiple birth (i.e., twins, triplets). We employed an automated data cleaning algorithm which excluded data observations of height and weight identified as biologically implausible. For the GWAS analysis, individuals without genetic data or who were flagged as being an outlier for any of the estimated phenotypes were excluded.
Replication	This study focuses on developing new statistical methods to model longitudinal traits in GWAS and detect age-varying genetic effects, so 'replication of findings' does not make sense in this context. However, we compared the cohorts to ensure 'replication' across cohorts for the longitudinal modeling and estimating the phenotypes aspect of the study. Additionally, we checked for evidence of heterogeneity in the genetic effects estimated across the cohorts in the meta-analysis to verify that there was consistency between the the cohorts.
Randomization	This study involves a population based cohort so randomization was not performed.
Blinding	No blinding is required because this study used data from the several cohort studies, which were observational and had no specific interventions.

Reporting for specific materials, systems and methods

We require information from authors about some types of materials, experimental systems and methods used in many studies. Here, indicate whether each material, system or method listed is relevant to your study. If you are not sure if a list item applies to your research, read the appropriate section before selecting a response.

Materials & experimental systems

n/a	Involved in the study
☒	☐ Antibodies
☒	☐ Eukaryotic cell lines
☒	☐ Palaeontology and archaeology
☒	☐ Animals and other organisms
☒	☐ Clinical data
☒	☐ Dual use research of concern
☒	☐ Plants

Methods

n/a	Involved in the study
☒	☐ ChIP-seq
☒	☐ Flow cytometry
☒	☐ MRI-based neuroimaging

Plants

Seed stocks	*Report on the source of all seed stocks or other plant material used. If applicable, state the seed stock centre and catalogue number. If plant specimens were collected from the field, describe the collection location, date and sampling procedures.*
Novel plant genotypes	*Describe the methods by which all novel plant genotypes were produced. This includes those generated by transgenic approaches, gene editing, chemical/radiation-based mutagenesis and hybridization. For transgenic lines, describe the transformation method, the number of independent lines analyzed and the generation upon which experiments were performed. For gene-edited lines, describe the editor used, the endogenous sequence targeted for editing, the targeting guide RNA sequence (if applicable) and how the editor was applied.*
Authentication	*Describe any authentication procedures for each seed stock used or novel genotype generated. Describe any experiments used to assess the effect of a mutation and, where applicable, how potential secondary effects (e.g. second site T-DNA insertions, mosiacism, off-target gene editing) were examined.*
